# Mortality risk attributable to high and low ambient temperature: a multicountry observational study

**DOI:** 10.1016/S0140-6736(14)62114-0

**Published:** 2015-07-25

**Authors:** Antonio Gasparrini, Yuming Guo, Masahiro Hashizume, Eric Lavigne, Antonella Zanobetti, Joel Schwartz, Aurelio Tobias, Shilu Tong, Joacim Rocklöv, Bertil Forsberg, Michela Leone, Manuela De Sario, Michelle L Bell, Yue-Liang Leon Guo, Chang-fu Wu, Haidong Kan, Seung-Muk Yi, Micheline de Sousa Zanotti Stagliorio Coelho, Paulo Hilario Nascimento Saldiva, Yasushi Honda, Ho Kim, Ben Armstrong

**Affiliations:** a Department of Medical Statistics, London School of Hygiene & Tropical Medicine, London, UK; b Department of Social and Environmental Health Research, London School of Hygiene & Tropical Medicine, London, UK; c Department of Epidemiology and Biostatistics, School of Population Health, University of Queensland, Brisbane, QLD, Australia; d Department of Pediatric Infectious Diseases, Institute of Tropical Medicine, Nagasaki University, Nagasaki, Japan; e Interdisciplinary School of Health Sciences, University of Ottawa, Ottawa, ON, Canada; f Department of Environmental Health, Harvard School of Public Health, Boston, MA, USA; g Institute of Environmental Assessment and Water Research (IDAEA), Spanish Council for Scientific Research (CSIC), Barcelona, Spain; h School of Public Health and Social Work, Queensland University of Technology, Brisbane, QLD, Australia; i Department of Public Health and Clinical Medicine, Umeå University, Umeå, Sweden; j Department of Epidemiology, Lazio Regional Health Service, Rome, Italy; k School of Forestry and Environmental Studies, Yale University, New Haven, CT, USA; l Department of Environmental and Occupational Medicine, National Taiwan University, Taipei, Taiwan; m Department of Public Health, National Taiwan University, Taipei, Taiwan; n Department of Environmental Health, Fudan University, Shanghai, China; o Graduate School of Public Health & Institute of Health and Environment, Seoul National University, Seoul, Republic of Korea; p Department of Pathology, School of Medicine, University of São Paulo, São Paulo, Brazil; q Faculty of Health and Sport Sciences, University of Tsukuba, Tsukuba, Japan

## Abstract

**Background:**

Although studies have provided estimates of premature deaths attributable to either heat or cold in selected countries, none has so far offered a systematic assessment across the whole temperature range in populations exposed to different climates. We aimed to quantify the total mortality burden attributable to non-optimum ambient temperature, and the relative contributions from heat and cold and from moderate and extreme temperatures.

**Methods:**

We collected data for 384 locations in Australia, Brazil, Canada, China, Italy, Japan, South Korea, Spain, Sweden, Taiwan, Thailand, UK, and USA. We fitted a standard time-series Poisson model for each location, controlling for trends and day of the week. We estimated temperature–mortality associations with a distributed lag non-linear model with 21 days of lag, and then pooled them in a multivariate metaregression that included country indicators and temperature average and range. We calculated attributable deaths for heat and cold, defined as temperatures above and below the optimum temperature, which corresponded to the point of minimum mortality, and for moderate and extreme temperatures, defined using cutoffs at the 2·5th and 97·5th temperature percentiles.

**Findings:**

We analysed 74 225 200 deaths in various periods between 1985 and 2012. In total, 7·71% (95% empirical CI 7·43–7·91) of mortality was attributable to non-optimum temperature in the selected countries within the study period, with substantial differences between countries, ranging from 3·37% (3·06 to 3·63) in Thailand to 11·00% (9·29 to 12·47) in China. The temperature percentile of minimum mortality varied from roughly the 60th percentile in tropical areas to about the 80–90th percentile in temperate regions. More temperature-attributable deaths were caused by cold (7·29%, 7·02–7·49) than by heat (0·42%, 0·39–0·44). Extreme cold and hot temperatures were responsible for 0·86% (0·84–0·87) of total mortality.

**Interpretation:**

Most of the temperature-related mortality burden was attributable to the contribution of cold. The effect of days of extreme temperature was substantially less than that attributable to milder but non-optimum weather. This evidence has important implications for the planning of public-health interventions to minimise the health consequences of adverse temperatures, and for predictions of future effect in climate-change scenarios.

**Funding:**

UK Medical Research Council.

## Introduction

Many epidemiological studies have provided evidence for the association between ambient temperature and mortality or morbidity outcomes.^1^, ^2^ Interest in this topic has increased after episodes of extreme weather and in response to reports about climate change.^3^, ^4^, ^5^

Although consensus exists among researchers that both extremely cold and extremely hot temperatures affect health, their relative importance is a matter of current debate and other details of the association remain unexplored. For example, little is known about the optimum temperatures that correspond to minimum effects for various health outcomes. Furthermore, most research has focused on extreme events and no studies have comparatively assessed the contribution of moderately high and low temperatures. The underlying physiopathological mechanisms that link exposure to non-optimum temperature and mortality risk have not been completely elucidated. Heat stroke on hot days and hypothermia on cold days only account for small proportions of excess deaths. High and low temperatures have been associated with increased risk for a wide range of cardiovascular, respiratory, and other causes, suggesting the existence of multiple biological pathways.^6^, ^7^, ^8^, ^9^ Ambient temperature represents an important risk factor and further investigation is needed to strengthen understanding of the associated health effects. This information is essential for planning of suitable public health interventions and for provision of reliable predictions for the effects of climate change.

Epidemiological studies of the topic face important challenges in modelling of temperature–health dependencies. First, the dose-response association, which is inherently non-linear, is also characterised by different lag periods for heat and cold—ie, excess risk caused by heat is typically immediate and occurs within a few days, while the effects of cold have been reported to last up to 3 or 4 weeks.^6^, ^7^ Second, the association is heterogeneous between populations because of acclimatisation, different adaptation responses, and variability in susceptibility factors.^10^, ^11^, ^12^ Modelling of such complex patterns needs a sophisticated statistical approach. Although studies have quantified the association in terms of relative risk (RR), few have given estimates of the attributable burden, either as absolute excess (numbers) or relative excess (fractions) of deaths.^13^, ^14^, ^15^, ^16^, ^17^, ^18^, ^19^ The evidence for the attributable risk of temperature is very often restricted to extreme events, especially heatwaves,^17^, ^18^ although few investigations have reported values from dose-response associations estimated in models with temperature as a continuous variable.^13^, ^14^

We aimed to quantify total mortality burden attributable to non-optimum ambient temperature, and the relative contributions from heat and cold and from moderate and extreme temperatures. We based our analysis on recent advances in statistical modelling to account for the complex and heterogeneous temperature–mortality dependency.

## Methods

### Study design and data

We collected time-series daily data, including mortality, weather variables, and air pollution measures, from 384 locations in 13 countries: Australia (three cities, 1988–2009), Brazil (18 cities, 1997–2011), Canada (21 cities, 1986–2009), China (15 cities, 1996–2008), Italy (11 cities, 1987–2010), Japan (47 prefectures, 1985–2012), South Korea (seven cities, 1992–2010), Spain (51 cities, 1990–2010), Sweden (one county, 1990–2002), Taiwan (three cities, 1994–2007), Thailand (62 provinces, 1999–2008), UK (ten regions, 1993–2006), and USA (135 cities, 1985–2009). Mortality was represented by daily counts of deaths for either all causes or, where not available, non-external causes only (International Classification of Diseases [ICD]-9 0-799, ICD-10 A00-R99). We chose mean daily temperature as the exposure index, calculated from central monitor stations, either as the average between maximum and minimum values or the 24 h average. We did a sensitivity analysis by modifying the modelling choices, replacing all-cause with non-external mortality, and controlling for air pollution and humidity in the subset of countries that provided such information. The appendix contains details of the exact study periods, further information on data collection, additional results, and results from the sensitivity analysis.

### Statistical analysis

We did all analysis with R software (version 3.0.3) using the packages *dlnm* and *mvmeta*. The code is available on request, and a reproducible example is included at the personal website of the first author. We first applied a standard time-series quasi-Poisson regression separately in each location to derive estimates of location-specific temperature–mortality associations, reported as RR. Specific tutorials explain the technical details and terminology.^20^ Briefly, this first-stage regression included a natural cubic B-spline of time with 8 degrees of freedom per year to control for seasonal and long-term trends, and an indicator of day of the week. We modelled the association with temperature using a distributed lag non-linear model.^21^ This class of models can describe complex non-linear and lagged dependencies through the combination of two functions that define the conventional exposure-response association and the additional lag-response association, respectively. The lag-response association represents the temporal change in risk after a specific exposure, and it estimates the distribution of immediate and delayed effects that cumulate across the lag period. Specifically, we modelled the exposure-response curve with a quadratic B-spline with three internal knots placed at the 10th, 75th, and 90th percentiles of location-specific temperature distributions, and the lag-response curve with a natural cubic B-spline with an intercept and three internal knots placed at equally spaced values in the log scale. We extended the lag period to 21 days to include the long delay of the effects of cold and to exclude deaths that were advanced by only a few days (harvesting effect). We tested these modelling choices in sensitivity analysis.

We then reduced the association to the overall temperature–mortality association, cumulating the risk during the lag period.^22^ This step reduces the number of parameters to be pooled in the second-stage meta-analysis, and preserves the complexity of the estimated dependency, thus avoiding unnecessary simplification.

We pooled the estimated location-specific overall cumulative exposure-response associations using a multivariate meta-analytical model.^22^, ^23^ Previous studies have reported how climatological, socioeconomic, demographic, and infrastructural factors have a role in modification of the association between temperature and mortality.^10^ To account for the main features of such effect modification, we included location-specific average temperature, temperature range, and indicators for country as meta-predictors in a multivariate meta-regression. We tested these effects through a multivariate Wald test. We tested residual heterogeneity and reported it by the multivariate extension of Cochran *Q* test and *I*^2^ statistic.^23^, ^24^

We used the fitted meta-analytical model to derive the best linear unbiased prediction of the overall cumulative exposure-response association in each location. The best linear unbiased prediction represents a trade-off between the location-specific association provided by the first-stage regression and the pooled association. This approach allows areas with small daily mortality counts or short series, usually characterised by very imprecise estimates, to borrow information from larger populations that share similar characteristics.^13^, ^23^, ^25^

The minimum mortality temperature, which corresponds to a minimum mortality percentile between the first and the 99th percentiles, was derived from the best linear unbiased prediction of the overall cumulative exposure-response association in each location. We referred to this value as the optimum temperature, and deemed it the reference for calculating the attributable risk by re-centring the quadratic B-spline that models the exposure-response. For each day of the series, in each location, we used the overall cumulative RR corresponding to each day's temperature to calculate the attributable deaths and fraction of attributable deaths in the next 21 days, using a previously described method.^26^

The total attributable number of deaths caused by non-optimum temperatures is given by the sum of the contributions from all the days of the series, and its ratio with the total number of deaths provides the total attributable fraction. We calculated the components attributable to cold and heat by summing the subsets corresponding to days with temperatures lower or higher than the minimum mortality temperature. We further separated these components into moderate and extreme contributions by defining extreme cold and heat as temperatures lower than the 2·5th location-specific percentile (extreme cold) and higher than the 97·5th location-specific percentile (extreme heat). These cutoffs are consistent with previous definitions of extreme weather, such as heatwaves.^7^, ^14^, ^18^, ^19^ We defined moderate temperatures as the ranges between the optimum temperature and these cutoffs. We defined other ranges using cutoffs at the 10th, 25th, 50th, 75th, and 90th percentiles.

We calculated empirical CIs (eCIs) using Monte Carlo simulations, assuming a multivariate normal distribution of the best linear unbiased predictions of the reduced coefficients. We reported algebraic equations and details elsewhere,^26^ and they are summarised in the appendix.

### Role of the funding source

The funder of the study had no role in study design, data collection, data analysis, data interpretation, or writing of the report. The corresponding author had full access to all the data in the study and had final responsibility for the decision to submit for publication.

## Results

Table 1 shows the descriptive statistics from each country. The dataset included 74 225 200 deaths. As expected, the populations in different countries experienced a broad range of temperatures, with country-specific averages ranging from 6·5°C in Canada to 27·6°C in Thailand. These temperatures are illustrative of regions characterised by different climates: from cold countries (Canada, Sweden, and to a lesser extent UK), through temperate latitudes in the Mediterranean (Spain and Italy), east Asia (South Korea and Japan), and southern-hemisphere areas (Australia), to tropical and subtropical areas (Brazil, Taiwan, and Thailand). Other large countries (China and USA) included locations with more heterogeneous climates.

**Table 1:** Descriptive statistics by country

	**Locations**	**Study period**	**Total deaths**	**Temperature (°C)**
Australia	3	1988–2009	1 177 950	18·1 (15·7–20·3)
Brazil	18	1997–2011	3 401 136	24·2 (17·7–27·4)
Canada	21	1986–2009	2 521 586	6·5 (2·6–10·7)
China	15	1996–2008	950 130	15·1 (7·4–23·7)
Italy	11	1987–2010	820 390	15·4 (12·2–18·4)
Japan	47	1985–2012	26 893 197	15·3 (9·1–23·1)
South Korea	7	1992–2010	1 726 938	13·7 (12·5–14·9)
Spain	51	1990–2010	3 479 910	15·5 (10·9–21·6)
Sweden	1	1990–2002	190 092	7·5 (7·5–7·5)
Taiwan	3	1994–2007	765 893	24·0 (23·2–25·2)
Thailand	62	1999–2008	1 827 853	27·6 (25·1–29·3)
UK	10	1993–2006	7 573 716	10·4 (9·5–11·7)
USA	135	1985–2006	22 896 409	14·9 (7·9–25·5)

Temperatures are mean location-specific temperature (range).

**Figure 1:**
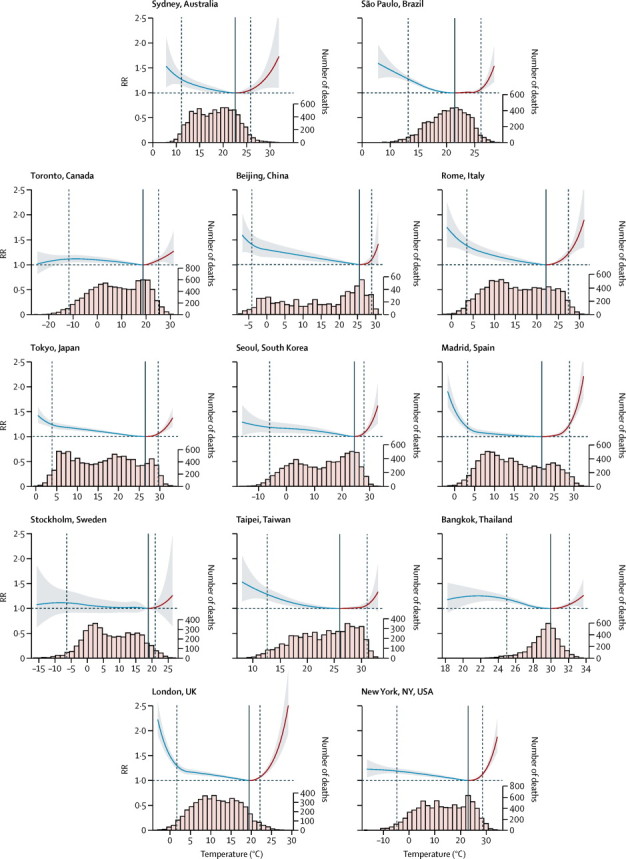
Overall cumulative exposure–response associations in 13 cities Exposure–response associations as best linear unbiased prediction (with 95% empirical CI, shaded grey) in representative cities of the 13 countries, with related temperature distributions. Solid grey lines are minimum mortality temperatures and dashed grey lines are the 2·5th and 97·5th percentiles. RR=relative risk.

Figure 1 shows overall cumulative exposure-response curves (best linear unbiased predictions) for 13 cities selected to represent each country, with the corresponding minimum mortality temperature and the cutoffs to define extreme temperatures. The corresponding graphs for all 384 locations are reported in the appendix. The temperature distributions emphasise how the hot temperature range, although characterised by a high RR, consists of only a small proportion of days. The median minimum mortality percentile ranges were at about the 80th and 90th percentiles for most countries, with the exception of the tropical and subtropical areas of Brazil, Taiwan, and Thailand, where it seemed to be near the 60th percentile (table 2). Risk increases slowly and linearly for cold temperatures below the minimum mortality temperature, although some locations (eg, London and Madrid) showed a higher increase for extreme cold than did the others. By contrast, risk generally escalated quickly and non-linearly at high temperatures.

**Table 2:** Attributable mortality by country

	**Minimum mortality percentile**	**Total**	**Cold**	**Heat**
Australia	83th	6·96% (4·27 to 9·51)	6·50% (3·91 to 8·94)	0·45% (0·20 to 0·70)
Brazil	60th	3·53% (3·00 to 4·01)	2·83% (2·34 to 3·30)	0·70% (0·45 to 0·93)
Canada	81st	5·00% (3·83 to 6·07)	4·46% (3·39 to 5·48)	0·54% (0·39 to 0·66)
China	83rd	11·00% (9·29 to 12·47)	10·36% (8·72 to 11·77)	0·64% (0·47 to 0·79)
Italy	79th	10·97% (8·03 to 13·43)	9·35% (6·59 to 11·72)	1·62% (1·24 to 1·98)
Japan	86th	10·12% (9·61 to 10·56)	9·81% (9·32 to 10·22)	0·32% (0·27 to 0·36)
South Korea	89th	7·24% (4·45 to 9·73)	6·93% (4·12 to 9·44)	0·31% (0·15 to 0·45)
Spain	78th	6·52% (5·82 to 7·16)	5·46% (4·79 to 6·07)	1·06% (0·96 to 1·16)
Sweden	93rd	3·87% (−6·20 to 12·93)	3·69% (−6·31 to 12·61)	0·18% (−0·47 to 0·65)
Taiwan	62nd	4·75% (3·26 to 6·06)	3·89% (2·50 to 5·31)	0·86% (0·12 to 1·50)
Thailand	60th	3·37% (3·06 to 3·63)	2·61% (2·31 to 2·88)	0·76% (0·65 to 0·86)
UK	90th	8·78% (8·00 to 9·54)	8·48% (7·72 to 9·25)	0·30% (0·25 to 0·36)
USA	84th	5·86% (5·50 to 6·17)	5·51% (5·17 to 5·82)	0·35% (0·30 to 0·39)
Total	81st	7·71% (7·43 to 7·91)	7·29% (7·02 to 7·49)	0·42% (0·39 to 0·44)

Attributable mortality computed as total and as separate components for cold and heat. Data are median percentile or % (95% empirical CI).

Results from our multivariate meta-regression suggest that, although significant, residual heterogeneity is low after country indicators, average temperature, and temperature range had been included as meta-predictors, with an *I*^2^ of 36·3%. Although all three predictors significantly modify the temperature–mortality association, either in single-predictor or full models, the country indicators account for a much higher proportion of heterogeneity than do average temperature or temperature range (appendix).

The main results (table 2) were the estimated attributable fraction calculated as total and as separated components caused by cold and hot temperatures in each country (see appendix for location-specific figures). Overall, the total fraction of deaths caused by both heat and cold was 7·71% (95% eCI 7·43–7·91), although this fraction varied substantially between countries, with the highest attributable risk in Italy, China, and Japan, and the lowest estimates in Thailand, Brazil, and Sweden (table 2). Although the CI for Sweden was not significant, the results seemed more likely to be caused by the small dataset than by a different pattern. Cold was responsible for most of the burden (total estimate 7·29%, 95% eCI 7·02–7·49%), while the fraction attributable to heat was small (0·42%, 0·39–0·44). This difference was mainly caused by the high minimum-mortality percentile, with most of the mean daily temperatures being lower than the optimum value.

**Figure 2:**
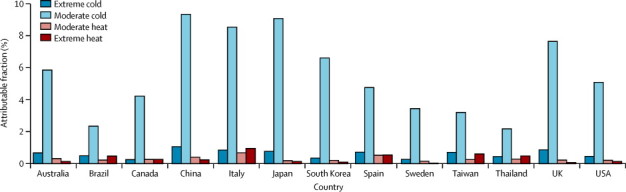
Fraction of all-cause mortality attributable to moderate and extreme hot and cold temperature by country Extreme and moderate high and low temperatures were defined with the minimum mortality temperature and the 2·5th and 97·5th percentiles of temperature. distribution as cutoffs.

The attributable risk can be separated into components related to moderate and extreme temperatures (figure 2). The appendix contains estimates for different temperature percentile ranges. In all countries, most of the mortality risk attributable to temperature was related to moderate cold, with an overall estimate of 6·66% (95% eCI 6·41–6·86). Extreme temperatures (either cold or hot) were responsible for a small fraction, corresponding to 0·86% (0·84–0·87%). These results are consistent with the exposure-response associations and temperature distributions in figure 1. Although the range corresponding to moderate cold had a comparatively low RR, it included the most days in the series. Our sensitivity analysis suggested that our results were not dependent on modelling assumptions (appendix).

## Discussion

Our findings show that temperature is responsible for advancing a substantial fraction of deaths, corresponding to 7·71% of mortality in the selected countries within the study period. Most of this mortality burden was caused by days colder than the optimum temperature (7·29%), compared with days warmer than the optimum temperature (0·42%). Furthermore, most deaths were caused by exposure to moderately hot and cold temperatures, and the contribution of extreme days was comparatively low, despite increased RRs. The study was based on the largest dataset ever collected to assess temperature–health associations, and included more than 74 million deaths from 13 countries (panel). The analysis of data from 384 locations provides evidence for temperature-related mortality risk in a wide range of climates and populations with different demographic, socioeconomic, and infrastructural characteristics. A strength of the study was the application of new, flexible statistical models to characterise the temperature-mortality association and pool estimates across locations. In particular, while previous studies relied on simplification of the exposure-response or lag structure, the approach we used here enabled us to estimate and pool non-linear and delayed dependencies and to identify the temperature of minimum mortality.

Panel **Research in context**

**Systematic review**

We searched the literature to identify articles that reported estimates of the effect of non-optimum ambient temperature on mortality and used attributable risk measures as the main effect summary. We searched PubMed using combinations of the terms “temperature” or “heat” or “cold”, and “mortality” or “death*”, and “attributable” or “impact”. We searched papers written in English from inception to Dec 31, 2013. We then manually selected relevant articles by reading the abstracts. Although several studies^13^, ^14^, ^15^, ^16^, ^17^, ^18^, ^19^ reported estimates of attributable risk, they used different definitions of summary measures and used various designs and analytical methods, which made the comparison difficult. Most of these investigations focused on heat-related health effects, and few assessed the attributable component caused by cold temperatures. More importantly, these studies restricted their assessments to single cities or countries, and no study has so far provided a comprehensive assessment across populations exposed to different climates by use of consistent statistical approaches.

**Interpretation**

We report that non-optimum ambient temperature is responsible for substantial excess in mortality, with important differences between countries. Although most previous research has focused on heat-related effects, most of the attributable deaths were caused by cold temperatures. Despite the attention given to extreme weather events, most of the effect happened on moderately hot and moderately cold days, especially moderately cold days. This evidence is important for improvements to public health policies aimed at prevention of temperature-related health consequences, and provides a platform to extend predictions on future effects in climate-change scenarios.

Comparison with previous studies that reported data for attributable deaths is limited by several factors, particularly the variation in study designs and modelling approaches and the use of alternative definitions of attributable risk measures. Findings from studies that focused on specific events or periods with extreme temperatures suggest a mortality increase of 8·9–12·1% during heat waves and 12·8% during cold spells.^16^, ^17^ Investigators who extended the analysis to the whole summer season report estimates of 1·6–2·0% for attributable mortality caused by heat.^13^, ^19^ Studies that include attributable risk measures for whole-year mortality, and thus adopt a comparable denominator, report values close to ours: Hajat and colleagues reported that the all-cause mortality attributable to heat was between 0·37% and 1·45% in three European cities,^14^ and Carson and colleagues^15^ estimated that 5·4% of deaths were attributable to cold but none to heat in London.

Various underlying mechanisms have been postulated to explain the increased mortality risk associated with exposure to high and low ambient temperature. Physiological effects leading to heat-related deaths are not well known yet, and probably vary for different mortality causes. In the case of the association of heat with cardiovascular mortality, the cause accounting for the greatest burden, acute events seem to be triggered when the body exceeds its thermoregulatory threshold, after changes in heart rate, blood viscosity and coagulability, reductions in cerebral perfusion, and attenuated vasoconstrictor responsiveness.^27^ Heat also increases mortality risk for other causes: a suggested mechanism is through the alteration of fluid and electrolytic balance in people affected by chronic diseases or in people with impaired responsiveness to environmental conditions.^1^, ^8^ These sudden physiological responses are consistent with the steep, supralinear increase in risk above the optimum temperature (figure 1, appendix), which was associated with a comparatively high burden attributable to extremely high temperature. The biological processes that underlie cold-related mortality mainly have cardiovascular and respiratory effects. Exposure to cold has been associated with cardiovascular stress by affecting factors such as blood pressure and plasma fibrinogen, vasoconstriction and blood viscosity, and inflammatory responses.^28^, ^29^ Similarly, cold induces bronchoconstriction and suppresses mucociliary defences and other immunological reactions, resulting in local inflammation and increased risk of respiratory infections.^30^ These physiological responses can persist for longer than those attributed to heat,^28^ and seem to produce mortality risks that follow a smooth, close-to-linear response, with most of the attributable risk occurring in moderately cold days.

Some limitations must be acknowledged. First, although this investigation includes populations with markedly different characteristics and living in a wide range of climates, the findings cannot be interpreted as globally representative. We did not include entire regions, such as Africa or the Middle East, and the assessment was mainly restricted to urban populations. Although our results suggest substantial intercountry variation in attributable risk for both heat and cold, the analysis did not characterise these differences to identify determinants of susceptibility or resilience to the effects of temperature. These limitations can be addressed in future research by extension of the dataset to populations living in other regions, and by collection of standardised measures of meta-variables for location-specific characteristics to be included in the second-stage meta-regression. Results from these analyses would complement the evidence provided in this study.

We identified a substantial effect of heat and cold on mortality, with attributable figures that varied by country. The optimum temperature at which the risk is lowest was well above the median, and seemed to be increased in cold regions. Cold was responsible for a higher proportion of deaths than was heat, while moderate hot and cold temperatures represented most of the total health burden. Research on the association between human health and ambient temperature has so far focused mainly on the effects of extreme heat, and public health plans have implemented policies and interventions designed almost exclusively for heatwave periods. Our results suggest that public-health policies and adaptation measures should be extended and refocused to take account of the whole range of effects associated with temperature, although further research is needed to clarify how much of the excess mortality related to each component is preventable. Our study also provides a platform to improve and extend predictions of the effects of climate change; our findings emphasise how a comprehensive assessment is needed to provide an appropriate estimate of the health consequences of various climate-change scenarios.
