# Supplementary material for: Mortality risk attributable to high and low ambient temperature: a multicountry observational study
Source: Lancet. 2015 Jul 25;386(9991):369–75. doi: 10.1016/S0140-6736(14)62114-0 (PMC4521077; doi:10.1016/S0140-6736(14)62114-0)
Supplement: Supplementary appendix [file mmc1.pdf]

THE LANCET

Supplementary appendix

This appendix formed part of the original submission and has been peer reviewed. We post it as supplied by the authors.

Supplement to: Gasparrini A, Guo Y, Hashizume M, et al. Mortality risk attributable to high and low ambient temperature: a multicountry observational study. *Lancet* 2015; published online May 21. http://dx.doi.org/10.1016/S0140-6736(14)62114-0.

Online appendix of the article:
Mortality risk attributable to high and low ambient temperature: a multi-country study

Additional information on data collection

Australia

We collected data from Melbourne, Sydney and Brisbane between 1st of January 1988 and 31st of May 2009. Daily mortality, obtained from the Australian Bureau of Statistics, is represented by counts of deaths for non-external causes only (ICD-9: 0-799; ICD-10: A00-R99). Mean daily temperature (in ˚C) and relative humidity (in %), computed as the 24-hour average based on hourly measurements, were obtained from the Australian Bureau of Meteorology. We selected all available meteorological stations located within ≤30 km of each city's Central Business District (CBD) (7 stations in Brisbane, 7 stations in Melbourne and 11 stations in Sydney). We calculated the daily averages of climatic variables using all records from meteorological stations in each city. When there was a missing value (≤1.3%) for a particular meteorological station, observations recorded from the remaining weather stations were used to compute the daily average values. In total, missing data amount for 0.16% and 0.00% of the mortality and temperature series, respectively. These data were used and described in previous publications [1,2]

Brazil

We collected data from 18 cities (see full list in Table S1 below) between 1st of January 1997 and 31st of December 2011. Daily mortality, obtained from the Ministry of Health, Brazil, is represented by counts of deaths for non-external causes only (ICD-9: 0-799; ICD-10: A00-R99). Mean daily temperature (in ˚C) and relative humidity (in %), computed from the 24-h average of hourly measurements) were obtained from the National Institute of Meteorology of Brazil. A single weather station located within the urban area was selected for each city. In total, missing data amount for 1.85% and 3.36% of the mortality and temperature series, respectively.

Canada

We collected data from 20 census metropolitan areas (CMA) and the city of Hamilton (see full list in Table S2 below) between 1st of January 1986 and 31st of December 2009. Daily mortality, obtained from Statistics Canada through access to the Canadian Mortality Database, is represented by counts of deaths for all causes. Mean daily temperature (in ˚C) and relative humidity (in %), computed as the 24-hour average based on hourly measurements, were obtained from Environment Canada. A single weather station was selected for each city using the airport monitoring station located closest to the CMA centre. Measures of ozone (O_3, in ppb), nitrogen dioxide (NO_2, in ppb) and particles ($PM_{2.5}$, in ppb) were available in the same period from the National Air Pollution Surveillance (NAPS) network of Environment Canada. Daily level of pollutants were computed as the 24-hour mean based on hourly measurements in different stations, and then averaged across stations with no missing data, with an average of 4 stations per city. In total, missing data amount for 1.98% and 0.91% of the mortality and temperature series, respectively. These data were used and described in previous publications.[3,4]

China

We collected data from the city of Anshan (1st of January 2004 to 31st of December 2006), Beijing (1st of January 2007 to 30th of September 2008), Fuzhou (1st of January 2004 to 31st of December 2006), Guangzhou (1st of January 2007 to 31st of December 2008), Hong Kong (1st of January 1996 to 31st of December 2002), Hangzhou (1st of January 2002 to 29th of December 2004), Lanzhou (1st of January 2004 to 31st of December 2008), Nanjing (1st of January 2007 to 31st of December 2010), Shanghai (1st of January 2001 to 31st of December 2004), Shenyang (1st of January 2005 to 31st of December 2008), Suzhu (1st of January 2005 to 31st of December 2008), Taiyuan (1st of January 2004 to 31st of December 2008), Tianjin(1st of January 2005 to 31st of December 2008), Wuhan (1st of January 2003 to 31st of December 2005), Wulumqi (1st of January 2006 to 31st of December 2007), and Xian (1st of January 2004 to 31st of December 2008). Daily mortality is represented by counts of deaths for non-external causes only (ICD-9: 0-799; ICD-10: A00-R99). Mean daily temperature (in ˚C) and relative humidity (in %) were computed as the 24-hour average based on hourly measurements.

Measures of nitrogen dioxide (NO_2, in μg/m^3), particles (PM10, in in μg/m^3) and sulphur dioxide (SO2, in in μg/m^3) were available in the same period. Daily level of pollutants were computed as the 24-hour mean based on hourly measurements. In total, missing data amount for 0.54% and 0.33% of the mortality and temperature series, respectively.

Italy

We collected data from the city of Bari (1st of January 1996 to 31st of December 2007), Bologna (1st of January 1996 to 31st of December 2010), Brescia (1st of January 1993 to 31st of December 2003), Civitavecchia (1st of January 1996 to 31st of December 2006), Frosinone (1st of January 1995 to 31st of December 2006), Genova (1st of January 1999 to 31st of December 2007), Latina (1st of January 1995 to 31st of December 2006), Palermo (1st of January 1997 to 31st of December 2001), Roma (1st of January 1987 to 31st of December 2010), Torino (1st of January 1991 an to 1st of December 1999), and Viterbo (1st of January 1995 to 1st of December 2006). Daily mortality, obtained from local mortality registries and from the rapid mortality surveillance system operational since 2004, is represented by counts of deaths for all causes. Mean daily temperature (in ˚C) and relative humidity (in %), computed as the 24-h average based on 6-h measurements, were obtained from the Meteorological Service of the Italian Air Force. A single weather station was selected for each city, using the airport monitoring station located closest to the city centre. In total, missing data amount for 1.95% and 3.30% of the mortality and temperature series, respectively. These data were used and described in previous publications.[5,6]

Japan

We collected data from 47 prefectures (see full list in Table S2 below) between 1st of January 1985 and 31st of December 2012. Daily mortality, obtained from computerized death certificate data from the Ministry of Health, Labour and Welfare, Japan, is represented by counts of deaths for all causes and for non-external causes only (ICD-9: 0-799; ICD-10: A00-R99). Mean daily temperature (in ˚C) and relative humidity (in %), computed as the 24-hour average based on hourly measurements, were obtained from the Japan Meteorology Agency. A single weather station located within the urban area of the capital city was selected for each prefecture. In total, missing data amount for 0.00% and 0.04% of the mortality and temperature series, respectively.

South Korea

We collected data from 7 cities (see full list in Table S2 below) between 1st of January 1992 and 31st of December 2010. Daily mortality is represented by counts of deaths for all causes and for non-external causes only (ICD-9: 0-799; ICD-10: A00-R99). Mean daily temperature (in ˚C) and relative humidity (in %) were computed as the 24-hour average based on hourly measurements. Measures of carbon monoxide (CO, in ppb), ozone (O_3, in ppb), nitrogen dioxide (NO_2, in ppb), particles (PM10, in ppb) and sulphur dioxide (SO2, in ppb) were available in the period 1999-2010. Fine particles measures (PM2.5, in ppb) were available only for Seoul in 2005-2009. Daily level of pollutants were computed as the 24-hour mean based on hourly measurements. In total, missing data amount for 3.77% and 3.77% of the mortality and temperature series, respectively.

Spain

We collected data from the 51 capital cities (see full list in Table S2 below) between 1st of January 1990 and 31st of December 2010. Daily mortality, obtained from Spain National Institute of Statistics, is represented by counts of deaths for all causes. Mean daily temperature (in ˚C), computed as the 24-hour average based on hourly measurements, was obtained from Spain National Meteorology Agency. A single weather station, located within the urban area or at the near airport, was selected for each city. Single-day missing values were imputed as the average of the days before and after. For periods longer than two days no imputation was done. In total, missing data amount for 0.00% and 2.00% of the mortality and temperature series, respectively. These data were used and described in previous publications.[7]

Sweden

We collected data from the county of Stockholm between 1st of January 1990 and 31st of December 2002. Daily mortality, obtained from the Swedish Cause of Death Register at the Swedish National Board of Health and

Welfare, is represented by counts of deaths for non-external causes only (ICD-9: 0-799; ICD-10: A00-R99). Mean daily temperature (in ˚C) and relative humidity (in %), computed as the 24-hour average based on hourly measurements, were obtained from the Environment and Health Administration. A single weather station, located at Torkel Knutssongatan in Central Stockholm, was selected. Measures ozone (O_3, in ppb) and nitrogen oxides (NO_x, in ppb) were available in the same period. Daily level of pollutants were computed as the 24-hour mean based on hourly measurements. In total, missing data amount for 0.00% and 6.59% of the mortality and temperature series, respectively. These data were used and described in previous publications.[8,9]

Taiwan

We collected data in Kaohsiung, Taipei and Taichung between 1st of January 1994 and 31st of December 2007. Daily mortality is represented by counts of deaths for all causes and for non-external causes only (ICD-9: 0-799; ICD-10: A00-R99). Mean daily temperature (in ˚C) and relative humidity (in %) were computed as the 24-hour average based on hourly measurements. Measures of carbon monoxide (CO, in ppb), ozone (O_3, in ppb), nitrogen dioxide (NO_2, in ppb), particles (PM10, in ppb) and sulphur dioxide (SO2, in ppb) were available for the same period. Fine particles measures (PM2.5, in ppb) were available only in 2005-2007. Daily level of pollutants were computed as the 24-hour mean based on hourly measurements. Data were pooled from 1 meteorological station and 11 air quality monitoring stations in Kaohsiung, 2 meteorological station and 5 air quality monitoring stations in Taichung, and 3 meteorological station and 15 air quality monitoring stations in Taipei, respectively. In total, missing data amount for 0.03% and 0.00% of the mortality and temperature series, respectively.

Thailand

We collected data from 62 provinces (see full list in Table S2 below) between 1st of January 1999 and 31st of December 2008. Daily mortality, obtained from the Ministry of Public Health, Thailand, is represented by counts of deaths for non-external causes only (ICD-9: 0-799; ICD-10: A00-R99). Mean daily temperature (in ˚C) and relative humidity (in %), computed as the average between daily minimum and maximum, were obtained from the Meteorological Department, Ministry of Information and Communication Technology, Thailand. A total of 117 weather stations in 62 provinces, with at least one weather monitoring station in each province. In total, missing data amount for 0.00% and 6.10% of the mortality and temperature series, respectively. Humidity measurements were missing in at least 10% of days in 12 provinces.

UK

We collected data in 9 regions of England and in Wales (see full list in Table S2 below) between 1st of January 1993 and 31st of December 2006. Daily mortality, obtained from the Office of National Statistics, is represented by counts of deaths for all causes and for non-external causes only (ICD-9: 0-799; ICD-10: A00-R99). Mean daily temperature (in ˚C) and relative humidity (in %), computed from the 24-h average of hourly measurements) were obtained from the British Atmospheric Data Centre. An average of 29 stations contributed data to each regional series, from a minimum of 7 in London to a maximum of 44 in Wales. In total, missing data amount for 0.00% and 0.00% of the mortality and temperature series, respectively. These data were used and described in previous publications.[10,11]

USA

We collected data from 135 cities (see full list in Table S2 below) between 1st of January 1985 and 31st of December 2006. Daily mortality, obtained from the National Center for Health Statistics (NCHS), is represented by counts of deaths for non-external causes only (ICD-9: 0-799; ICD-10: A00-R99). Mean daily temperature (in ˚C, computed as the 24-hour average based on hourly measurements) and relative humidity (in %, computed from the 24-h average of hourly measurements of dew point temperature) were obtained from the National Climatic Data Center (NCDC) of the National Oceanic and Atmospheric Administration (NOAA). A single weather station was selected for each city in the land-based station data or NCDC, based on the proximity to the city's population centre. In 6 cities where multiple observations were missing from all the nearby monitors, hourly data from the Integrated Surface Database Lite of NCDC were converted in daily values. For 25 stations missing dew point data, dew point data were obtained from the nearest station with dew point data. In total, missing data amount for 0.32% and 1.89% of the mortality and temperature series, respectively. These data were used and described in previous publications.[12,13]

Additional information on the statistical methods

Details on the computation of the attributable risk

The mortality risk attributable to a temperature x_t for a given day t in the series is defined as the number $AN_{x,t}$ and fraction $AF_{x,t}$ of deaths experienced in the next L days, with L as the maximum lag period, defined by:

$$AF_{x,t} = 1 - \exp\left(-\sum_{l=0}^{L} \beta_{x_t,l}\right)$$

and

$$AN_{x,t} = AF_{x,t} \cdot \sum_{l=0}^{L} \frac{n_{t+l}}{L+1}$$

with $\sum \beta_{x_t,l}$ as the overall cumulative log-relative risk for temperature x_t in day t, and n_t as the number of deaths in day t. To be noted how the number of attributable deaths $AN_{x,t}$ is computed as the fraction of the average mortality in the future L days. The risk estimate $\sum \beta_{x_t,l}$ is obtained by the BLUP of the overall cumulative exposure-response association, re-centered on the temperature of minimum mortality (MMT). The MMT is therefore the counterfactual condition for the definition of the attributable risk. Therefore, the attributable risk can be interpreted as the excess deaths due to non-optimal temperature, if compared to a hypothetical situation in which temperature is constantly equal to the MMT.

The total attributable number of deaths AN_{tot} due to temperature is given by the sum of $AN_{x,t}$ for all the days t of the series, and its ratio with the total number of deaths provides the total attributable fraction AF_{tot}. The components attributable to cold and heat are computed by summing the subsets of $AN_{x,t}$ lower or higher than the temperature corresponding to the location-specific MMT. These components are further separated in moderate and extreme contributions by selecting low and high temperature cut-offs. Here extreme cold and heat are defined as the temperatures lower than the 2.5^{th} and higher than the 97.5^{th} city-specific percentiles, respectively. Moderate temperatures are instead defined as the ranges between the MMT and these cut-offs. Other cut-offs are used for a further stratification for different temperature ranges in Table S3.

The method is described in details in a previous publication.[14]

Additional tables

***Table S1*: Sensitivity analysis**

Computed on the fraction (%) attributable to temperature (total, heat, and cold components), by varying modelling choices, fitting the models to all-cause vs non-external mortality, and controlling for PM_{10}, ozone and humidity.

	**Total (%)**	**Cold (%)**	**Heat (%)**
Modelling choices (384 locations)			
Main model	7·71	7·29	0·42
Knots for exposure-response: 10th, 50th, and 90th	7·04	6·7	0·34
Knots for exposure-response: 10th, 25th, 75th and 90th	7·68	7·22	0·46
Cubic B-spline for exposure-response	7·51	7·08	0·43
Df for lag-response: 6	7·65	7·23	0·42
Lag period: 14 days	6·94	6·46	0·48
Lag period: 28 days	7·96	7·60	0·36
Df/year for seasonal control: 6	7·08	6·76	0·32
Df/year for seasonal control: 10	6·25	5·85	0·40
Outcome: all-cause or non-external mortality (67 locations)			
All-cause	9·58	9·25	0·33
Non-external	9·64	9·33	0·30
Control for PM_{10} (25 locations)			
Without PM_{10}	7·94	7·27	0·67
With PM_{10}	8·43	7·64	0·80
Control for ozone (24 locations)			
Without ozone	6·10	5·59	0·51
With ozone	6·34	5·86	0·49
Control for relative humidity (320 locations)			
Without relative humidity	7·82	7·43	0·39
With relative humidity	7·77	7·36	0·41

***Table S2*: Second-stage random-effects meta-regression models**

Significance test for predictors (p-value), multivariate Cochran Q test for heterogeneity (p-value), and I^2 statistic (%) in different multivariate random-effects meta-regression models.

**Model**	**Predictor**	**Test for predictor**	**Q test**	I^2
**Intercept-only**	-	-	<0.001	57.50%
**Single predictor**	Average temperature	<0.001	<0.001	54.10%
	Temperature range	<0.001	<0.001	53.60%
	Country	<0.001	<0.001	41.80%
**Full model**	Average temperature	<0.001	<0.001	36.30%
	Temperature range	<0.001		
	Country	<0.001		

***Table S3*: Attributable mortality fraction by country computed as separated components for heat and cold at different temperature percentiles ranges**

Country-specific fraction (%) of all-cause mortality attributable to temperature in each country, reported as separated components due to different temperature ranges, with 95% empirical confidence intervals.

		**Min ≤ t ≤ 2.5th**	**2.5th < t ≤ 10th**	**10th < t ≤ 25th**	**25th < t < 50th**	**50th ≤ t < 75th**	**75th ≤ t < 90th**	**90th ≤ t < 97.5th**	**97.5th ≤ t ≤ Max**
**Australia**	Heat (%)	0·00 (0·00 – 0·00)	0·00 (0·00 – 0·00)	0·00 (0·00 – 0·00)	0·00 (0·00 – 0·00)	0·00 (-0·01 – 0·01)	0·01 (-0·04 – 0·06)	0·13 (-0·03 – 0·30)	0·32 (0·19 – 0·42)
	Cold (%)	0·67 (0·49 – 0·83)	1·43 (0·99 – 1·82)	2·04 (1·27 – 2·73)	1·75 (0·85 – 2·65)	0·56 (0·05 – 1·07)	0·04 (-0·10 – 0·17)	0·00 (0·00 – 0·00)	0·00 (0·00 – 0·00)
**Brazil**	Heat (%)	0·00 (0·00 – 0·00)	0·00 (0·00 – 0·00)	0·00 (0·00 – 0·00)	0·00 (0·00 – 0·01)	0·09 (0·02 – 0·16)	0·21 (0·09 – 0·32)	0·18 (0·10 – 0·26)	0·22 (0·17 – 0·27)
	Cold (%)	0·49 (0·44 – 0·54)	0·89 (0·76 – 1·00)	0·90 (0·73 – 1·05)	0·47 (0·30 – 0·64)	0·08 (0·02 – 0·14)	0·00 (0·00 – 0·00)	0·00 (0·00 – 0·00)	0·00 (0·00 – 0·00)
**Canada**	Heat (%)	0·00 (0·00 – 0·00)	0·00 (0·00 – 0·00)	0·00 (0·00 – 0·00)	0·00 (0·00 – 0·00)	0·00 (0·00 – 0·00)	0·04 (0·01 – 0·07)	0·23 (0·15 – 0·32)	0·26 (0·21 – 0·31)
	Cold (%)	0·25 (0·17 – 0·32)	0·76 (0·58 – 0·92)	1·30 (1·00 – 1·58)	1·53 (1·12 – 1·91)	0·61 (0·41 – 0·78)	0·01 (0·00 – 0·02)	0·00 (0·00 – 0·00)	0·00 (0·00 – 0·00)
**China**	Heat (%)	0·00 (0·00 – 0·00)	0·00 (0·00 – 0·00)	0·00 (0·00 – 0·00)	0·00 (0·00 – 0·00)	0·00 (0·00 – 0·00)	0·01 (-0·02 – 0·04)	0·23 (0·12 – 0·32)	0·40 (0·32 – 0·47)
	Cold (%)	1·06 (0·96 – 1·15)	1·98 (1·73 – 2·19)	2·97 (2·54 – 3·35)	3·26 (2·67 – 3·81)	1·04 (0·71 – 1·34)	0·06 (-0·05 – 0·16)	0·00 (0·00 – 0·00)	0·00 (0·00 – 0·00)
**Italy**	Heat (%)	0·00 (0·00 – 0·00)	0·00 (0·00 – 0·00)	0·00 (0·00 – 0·00)	0·00 (0·00 – 0·00)	0·00 (0·00 – 0·00)	0·19 (0·09 – 0·30)	0·75 (0·52 – 0·97)	0·67 (0·56 – 0·77)
	Cold (%)	0·85 (0·69 – 0·98)	1·86 (1·43 – 2·23)	2·79 (2·03 – 3·44)	2·93 (1·82 – 3·86)	0·93 (0·36 – 1·43)	0·00 (0·00 – 0·01)	0·00 (0·00 – 0·00)	0·00 (0·00 – 0·00)
**Japan**	Heat (%)	0·00 (0·00 – 0·00)	0·00 (0·00 – 0·00)	0·00 (0·00 – 0·00)	0·00 (0·00 – 0·00)	0·00 (0·00 – 0·00)	0·01 (0·00 – 0·02)	0·12 (0·10 – 0·15)	0·18 (0·16 – 0·20)
	Cold (%)	0·77 (0·74 – 0·79)	1·79 (1·71 – 1·86)	2·88 (2·74 – 3·01)	3·09 (2·92 – 3·24)	1·18 (1·08 – 1·27)	0·10 (0·06 – 0·12)	0·00 (0·00 – 0·00)	0·00 (0·00 – 0·00)
**South Korea**	Heat (%)	0·00 (0·00 – 0·00)	0·00 (0·00 – 0·00)	0·00 (0·00 – 0·00)	0·00 (0·00 – 0·00)	0·00 (0·00 – 0·00)	0·00 (-0·01 – 0·01)	0·10 (0·01 – 0·19)	0·21 (0·13 – 0·27)
	Cold (%)	0·35 (0·22 – 0·46)	0·96 (0·60 – 1·28)	1·83 (1·17 – 2·38)	2·48 (1·54 – 3·27)	1·16 (0·57 – 1·73)	0·13 (-0·19 – 0·42)	0·02 (-0·13 – 0·15)	0·00 (-0·01 – 0·01)
**Spain**	Heat (%)	0·00 (0·00 – 0·00)	0·00 (0·00 – 0·00)	0·00 (0·00 – 0·00)	0·00 (0·00 – 0·00)	0·00 (0·00 – 0·00)	0·10 (0·07 – 0·13)	0·44 (0·38 – 0·50)	0·52 (0·49 – 0·55)
	Cold (%)	0·71 (0·67 – 0·75)	1·20 (1·08 – 1·30)	1·61 (1·43 – 1·78)	1·50 (1·24 – 1·74)	0·44 (0·31 – 0·56)	0·00 (0·00 – 0·00)	0·00 (0·00 – 0·00)	0·00 (0·00 – 0·00)
**Sweden**	Heat (%)	0·00 (0·00 – 0·00)	0·00 (0·00 – 0·00)	0·00 (0·00 – 0·00)	0·00 (0·00 – 0·00)	0·00 (0·00 – 0·00)	0·00 (0·00 – 0·00)	0·03 (-0·09 – 0·14)	0·15 (-0·39 – 0·53)
	Cold (%)	0·27 (-0·32 – 0·75)	0·70 (-0·73 – 1·85)	0·99 (-1·50 – 3·07)	0·99 (-2·53 – 4·03)	0·50 (-2·11 – 2·91)	0·25 (-1·20 – 1·47)	0·01 (-0·06 – 0·06)	0·00 (0·00 – 0·00)
**Taiwan**	Heat (%)	0·00 (0·00 – 0·00)	0·00 (0·00 – 0·00)	0·00 (0·00 – 0·00)	0·00 (-0·01 – 0·01)	0·08 (-0·14 – 0·29)	0·23 (-0·05 – 0·50)	0·29 (0·04 – 0·52)	0·25 (0·11 – 0·37)
	Cold (%)	0·71 (0·55 – 0·83)	1·30 (0·93 – 1·66)	1·31 (0·79 – 1·81)	0·56 (0·06 – 1·08)	0·02 (-0·07 – 0·11)	0·00 (0·00 – 0·00)	0·00 (0·00 – 0·00)	0·00 (0·00 – 0·00)
**Thailand**	Heat (%)	0·00 (0·00 – 0·00)	0·00 (0·00 – 0·00)	0·00 (0·00 – 0·00)	0·00 (0·00 – 0·00)	0·01 (0·00 – 0·03)	0·14 (0·10 – 0·19)	0·32 (0·27 – 0·37)	0·28 (0·24 – 0·31)
	Cold (%)	0·44 (0·41 – 0·47)	0·99 (0·93 – 1·04)	0·86 (0·76 – 0·96)	0·30 (0·19 – 0·40)	0·02 (-0·01 – 0·05)	0·00 (0·00 – 0·00)	0·00 (0·00 – 0·00)	0·00 (0·00 – 0·00)
**UK**	Heat (%)	0·00 (0·00 – 0·00)	0·00 (0·00 – 0·00)	0·00 (0·00 – 0·00)	0·00 (0·00 – 0·00)	0·00 (0·00 – 0·00)	0·00 (0·00 – 0·00)	0·07 (0·04 – 0·11)	0·22 (0·19 – 0·26)
	Cold (%)	0·86 (0·82 – 0·90)	1·27 (1·14 – 1·37)	2·04 (1·84 – 2·24)	2·73 (2·45 – 3·02)	1·42 (1·25 – 1·59)	0·16 (0·10 – 0·23)	0·00 (0·00 – 0·00)	0·00 (0·00 – 0·00)
**USA**	Heat (%)	0·00 (0·00 – 0·00)	0·00 (0·00 – 0·00)	0·00 (0·00 – 0·00)	0·00 (0·00 – 0·00)	0·01 (0·00 – 0·01)	0·02 (0·01 – 0·03)	0·11 (0·09 – 0·13)	0·21 (0·19 – 0·23)
	Cold (%)	0·45 (0·43 – 0·47)	1·06 (1·01 – 1·11)	1·57 (1·48 – 1·65)	1·61 (1·49 – 1·71)	0·71 (0·64 – 0·78)	0·10 (0·07 – 0·13)	0·01 (0·00 – 0·01)	0·00 (0·00 – 0·00)
**TOT**	Heat (%)	0·00 (0·00 – 0·00)	0·00 (0·00 – 0·00)	0·00 (0·00 – 0·00)	0·00 (0·00 – 0·00)	0·01 (0·00 – 0·01)	0·03 (0·03 – 0·04)	0·15 (0·13 – 0·16)	0·23 (0·22 – 0·24)
	Cold (%)	0·63 (0·61 – 0·64)	1·36 (1·32 – 1·39)	2·08 (2·01 – 2·14)	2·22 (2·13 – 2·30)	0·90 (0·85 – 0·95)	0·09 (0·07 – 0·11)	0·00 (0·00 – 0·01)	0·00 (0·00 – 0·00)

Table S4: **Attributable mortality fraction by location computed as total and as separated components for cold and heat**

Number of deaths, location-specific minimum mortality temperature (MMT) and percentile (MMP), and fraction (%) of all-cause mortality attributable to temperature in each location. The latter is reported as total and as separated components due to cold and heat, with 95% empirical confidence intervals.

Country	Location	Deaths	MMP	MMT	Overall (%)	Cold (%)	Heat (%)
Australia	Brisbane	191996	68	22·8	5·06	4·65	0·42
Australia	Melbourne	449092	90	22·4	6·48	5·99	0·49
Australia	Sydney	536862	83	22·6	8·04	7·6	0·44
Brazil	Belem	132910	41	26·7	2·13	1·06	1·07
Brazil	Belo Horizonte	464778	60	22·8	2·04	1·58	0·47
Brazil	Brasilia	114801	81	22·9	3·04	2·73	0·31
Brazil	Cuiaba	51336	75	28·1	2·45	2	0·45
Brazil	Curitiba	155276	80	21·3	4·99	4·68	0·31
Brazil	Fortaleza	202340	42	26·9	3·21	1·68	1·52
Brazil	Goiania	134263	46	24·2	2·26	1·12	1·14
Brazil	Joao Pessoa	53970	47	27·1	2·83	2·08	0·75
Brazil	Maceio	92525	60	25·6	2·74	2·27	0·47
Brazil	Manaus	98204	56	27·4	2·58	1·7	0·88
Brazil	Natal	80753	83	24·5	4·18	3·88	0·3
Brazil	Porto Alegre	215734	81	24·2	7·08	6·58	0·5
Brazil	Recife	263492	39	25·7	2·1	0·89	1·2
Brazil	Salvador	223545	78	27	3·19	2·96	0·23
Brazil	Sao Luis	81852	46	26·9	2·08	1·28	0·8
Brazil	Sao Paulo	916233	60	21·5	4·62	3·93	0·69
Brazil	Teresina	67174	67	28	3·48	2·85	0·62
Brazil	Vitoria	51950	75	26·8	3·17	2·79	0·38
Canada	Abbotsford	24745	79	16·4	2·19	1·98	0·21
Canada	Calgary	121137	82	14·7	3·72	3·51	0·21
Canada	Edmonton	141883	81	15·6	5·89	5·43	0·46
Canada	Halifax	65807	80	16·4	4·83	4·41	0·43
Canada	Hamilton	105812	79	18·2	3·88	3·36	0·52
Canada	Kingston	36322	79	18	5·23	4·67	0·56
Canada	Kitchener-Waterloo	65205	82	18·1	5·59	5·01	0·57
Canada	London Ontario	89695	80	18·5	4·76	4·16	0·6
Canada	Montreal	255487	81	18·9	3·22	2·54	0·68
Canada	Ottawa	126248	80	18·3	5·81	5·19	0·62
Canada	Regina	45728	82	16·6	5·95	5·46	0·49
Canada	Sudbury	37715	81	16·7	5·96	5·38	0·59
Canada	Saint John NB	41001	83	15·6	4·38	4·1	0·28
Canada	St. John's NFL	50285	90	16·5	4·76	4·28	0·48
Canada	Saskatoon	52741	82	16·1	5·66	5·13	0·53
Canada	Thunder Bay	32761	82	15·4	5·84	5·27	0·57
Canada	Toronto	632509	80	18·9	6·04	5·32	0·72
Canada	Victoria	78187	82	15·7	2·59	2·41	0·18

Canada	Vancouver	302493	82	16·7	4·42	4·11	0·31
Canada	Windsor	60532	78	20·2	4·61	4·06	0·55
Canada	Winnipeg	155293	81	17·2	6·07	5·53	0·54
China	Anshan	30076	82	24·2	12·31	11·62	0·69
China	Beijing	74786	80	25·5	12·07	11·32	0·75
China	Fuzhou	17142	80	27·6	9·95	9·19	0·76
China	Guangzhou	57721	82	28·7	10·2	9·53	0·68
China	Hong Kong	213860	82	28·6	7·74	7·53	0·22
China	Hangzhou	21743	84	27	11·29	10·58	0·71
China	Lanzhou	33877	90	20	12·11	11·88	0·22
China	Shanghai	172940	90	29·2	11·82	11·14	0·68
China	Shenyang	96588	80	22	12·17	11·39	0·78
China	Suzhu	49633	82	26·9	12·18	11·43	0·75
China	Taiyuan	43771	86	23·3	12·74	11·85	0·89
China	Tianjin	15857	83	25·6	11·91	10·87	1·03
China	Wuhan	62440	90	29·9	12·94	11·75	1·2
China	Wulumqi	12281	85	23·3	12·19	11·46	0·74
China	Xian	47415	83	24	12·3	11·47	0·83
Italy	Bari	30526	78	22·6	10·35	8·93	1·42
Italy	Bologna	58033	78	22·9	10·69	8·28	2·41
Italy	Brescia	20318	79	22	10·73	8·69	2·04
Italy	Civitavecchia	3912	78	23·1	9·96	8·19	1·77
Italy	Frosinone	3537	78	22·1	10·73	8·96	1·77
Italy	Genoa	71214	79	22·4	10·46	9·21	1·24
Italy	Latina	7976	78	22·6	9·55	7·56	1·99
Italy	Palermo	26865	79	24·5	9·55	8·31	1·23
Italy	Rome	522179	78	22·1	11·53	9·96	1·57
Italy	Turin	69544	79	19·8	8·62	6·85	1·77
Italy	Viterbo	6286	79	21·5	10·69	8·87	1·82
Japan	Aichi	1275798	83	25·6	11·11	10·62	0·49
Japan	Akita	331451	91	24·1	11·96	11·52	0·44
Japan	Aomori	367639	89	22	10·13	9·53	0·6
Japan	Chiba	1043841	91	27	9·39	9·23	0·16
Japan	Ehime	389884	86	26·6	8·49	8·3	0·19
Japan	Fukushima	520578	92	25·8	12·09	11·67	0·42
Japan	Fukui	196861	84	24·7	10·23	9·98	0·24
Japan	Fukuoka	1079937	81	25·3	9·54	9·12	0·41
Japan	Gifu	461978	89	27·3	10·01	9·66	0·35
Japan	Gunma	452208	86	25·2	10·65	10·36	0·29
Japan	Hokkaido	1225695	93	22·4	8·67	8·47	0·19
Japan	Hiroshima	650143	83	25·8	10·46	10·08	0·38
Japan	Hyogo	1179655	86	26·8	8·85	8·78	0·07
Japan	Ibaraki	636919	91	25·3	10·1	9·88	0·22
Japan	Ishikawa	266873	86	25·2	10·62	10·18	0·44
Japan	Iwate	359039	91	23·2	12·82	12·38	0·44
Japan	Kagawa	259957	81	25·4	11·36	10·87	0·49

Japan	Kanagawa	1410360	88	26·1	9·82	9·56	0·26
Japan	Kagoshima	488815	80	26·6	9·16	8·93	0·23
Japan	Kochi	233994	93	27·9	8·4	8·37	0·03
Japan	Kumamoto	455294	82	26·2	9·65	9·51	0·14
Japan	Kyoto	566646	82	25·6	10·55	10·13	0·42
Japan	Mie	429830	91	27·5	10·5	10·29	0·21
Japan	Miyagi	478243	92	24·4	12·3	12	0·3
Japan	Miyazaki	283834	87	27·1	9·99	9·72	0·27
Japan	Nagano	539233	90	24·8	11·69	11·36	0·33
Japan	Nagasaki	384449	81	25·5	9·24	9	0·24
Japan	Nara	292894	85	25·3	9·93	9·69	0·24
Japan	Niigata	614347	83	23·6	11·96	11·37	0·59
Japan	Oita	314390	91	27·4	9·92	9·67	0·25
Japan	Okayama	474479	90	28·1	10·97	10·85	0·12
Japan	Okinawa	218136	91	29·1	6·48	6·37	0·11
Japan	Osaka	1718353	82	26·2	9·2	8·85	0·35
Japan	Saga	221728	81	25·4	11·22	10·82	0·4
Japan	Saitama	1132794	84	24·8	12	11·51	0·49
Japan	Shiga	260846	88	26·1	10·16	10·04	0·11
Japan	Shimane	219027	85	24·6	10·04	9·86	0·18
Japan	Shizuoka	789815	91	27	10·83	10·61	0·22
Japan	Tokushima	221039	90	27·5	8·14	8·05	0·09
Japan	Tochigi	441327	90	25·6	10·79	10·4	0·39
Japan	Tokyo	2318083	87	26·5	9·06	8·7	0·36
Japan	Tottori	163483	85	25	10·67	10·3	0·37
Japan	Toyama	277171	83	23·8	10·74	10·36	0·39
Japan	Wakayama	287978	80	25·4	10·96	10·6	0·36
Japan	Yamagata	332903	90	24·4	11·97	11·61	0·36
Japan	Yamaguchi	416241	81	24·5	9·38	8·89	0·49
Japan	Yamanashi	209009	85	25·2	10·42	10·11	0·3
South Korea	Busan	340551	99	28·7	6·97	6·98	0
South Korea	Daegu	207086	90	26·4	5·4	5·12	0·29
South Korea	Daejeon	105049	85	24·1	8·27	7·71	0·56
South Korea	Gwangju	108222	90	25·9	7·37	6·89	0·48
South Korea	Incheon	193478	88	24·1	6·49	6·32	0·17
South Korea	Seoul	716638	86	24·5	8·03	7·59	0·44
South Korea	Ulsan	55914	91	25·9	5·92	5·68	0·25
Spain	A Coruna	75572	80	18·7	5·71	5·15	0·56
Spain	Albacete	34158	78	22	7·65	6·25	1·39
Spain	Alicante	51253	76	23·6	6·88	6	0·89
Spain	Almeria	41618	77	24·1	6·59	5·65	0·94
Spain	Avila	21147	80	18·5	7·67	6·34	1·33
Spain	Badajoz	37608	78	23·7	7·48	6·16	1·31
Spain	Barcelona	365724	73	21	6·78	6·11	0·67
Spain	Bilbao	82030	80	19·6	4·74	4·07	0·68
Spain	Burgos	41193	81	17·8	7·22	6·14	1·08

Spain	Caceres	22017	78	23·2	8·06	6·63	1·43
Spain	Cadiz	40682	78	22·8	6·42	5·27	1·15
Spain	Castellon	41632	76	23·3	6·69	5·82	0·88
Spain	Ceuta	9793	76	22·8	5·7	4·85	0·85
Spain	Cordoba	71562	77	25	8·4	7·07	1·33
Spain	Ciudad Real	22650	78	23·6	8·74	7·16	1·59
Spain	Cuenca	16498	78	20·6	7·63	6·02	1·61
Spain	Girona	30810	78	21	6·75	5·6	1·15
Spain	Granada	77716	78	23	7·56	6·19	1·37
Spain	Guadalajara	20115	78	20·4	7·71	6·12	1·59
Spain	Huelva	43169	78	23·6	6·72	5·74	0·98
Spain	Huesca	15670	79	21·2	6·84	5·53	1·31
Spain	Jaen	36427	78	24	7·22	5·82	1·4
Spain	Leon	44880	80	17·7	8·07	6·91	1·16
Spain	Lleida	37200	78	22·4	6·67	5·1	1·56
Spain	Logrono	33058	80	20·7	6·28	5·15	1·13
Spain	Lugo	35048	81	17·2	7·16	6·38	0·77
Spain	Madrid	576566	76	21·9	5·4	4	1·4
Spain	Malaga	116461	74	23·1	6·54	5·47	1·07
Spain	Melilla	8727	78	23·7	5·53	4·82	0·71
Spain	Murcia	77678	76	23·3	4·93	4·32	0·61
Spain	Ourense	38757	78	20·5	6·45	5·53	0·92
Spain	Oviedo	71913	80	18	6·65	6·05	0·6
Spain	Pamplona	56897	81	19·7	5·12	4·16	0·96
Spain	Palmas G. Canaria	85973	64	22·6	4·24	3·6	0·64
Spain	Palma Mallorca	83128	77	22·6	7·19	6·23	0·96
Spain	Pontevedra	31206	79	19	7·48	6·62	0·87
Spain	Salamanca	45440	79	19·2	6·56	5·48	1·08
Spain	Santander	58362	78	18·8	5·01	4·35	0·66
Spain	Tenerife	52474	71	23·7	4·4	3·67	0·73
Spain	Segovia	17095	80	19·8	7·48	6·14	1·34
Spain	Sevilla	177514	77	25·7	8·89	7·43	1·46
Spain	Soria	12594	80	18·3	7·23	5·95	1·28
Spain	San Sebastian	66047	81	18·5	4·02	3·26	0·75
Spain	Tarragona	26417	77	23·8	6·37	5·13	1·24
Spain	Teruel	11995	78	19·3	7·31	5·6	1·71
Spain	Toledo	31042	77	23·4	7·1	5·67	1·43
Spain	Valencia	214073	78	24	7·81	7·03	0·78
Spain	Vitoria	38581	81	17·8	6·18	5·33	0·85
Spain	Valladolid	67795	79	20	7·98	6·75	1·23
Spain	Zamora	20858	79	20·3	7·44	6·16	1·28
Spain	Zaragoza	143087	78	22·6	6·14	4·81	1·33
Sweden	Stockholm	190092	93	18·9	3·87	3·69	0·18
Taiwan	Kaohsiung	212330	43	25·4	4·1	2·74	1·36
Taiwan	Taipei	390749	63	26	5·24	4·53	0·71
Taiwan	Taichung	162814	62	26·3	4·43	3·9	0·54

Thailand	Amnat Charoen	10191	56	27·8	3·15	2·4	0·75
Thailand	Ayutthaya	24630	56	28·9	3·3	2·38	0·92
Thailand	Bangkok	242841	64	29·9	2·87	2·1	0·77
Thailand	Buri Ram	31550	63	28·2	3·28	2·64	0·64
Thailand	Chachoengsao	19972	74	28·2	3·97	3·31	0·66
Thailand	Chumphon	11974	65	29·4	2·93	2·24	0·69
Thailand	Chon Buri	46481	65	29	2·69	1·98	0·72
Thailand	Chiang Mai	76294	69	27·9	3·58	2·95	0·63
Thailand	Chiang Rai	52925	74	27·4	3·87	3·25	0·61
Thailand	Chanthaburi	21381	60	27·9	3·3	2·69	0·61
Thailand	Chaiyaphum	28116	59	28·6	3·1	2·39	0·71
Thailand	Kalasin	30249	70	29·3	3·15	2·55	0·6
Thailand	Khon Kaen	59977	55	27·9	3·62	2·53	1·09
Thailand	Kamphaeng Phet	13792	65	27·9	3·51	2·81	0·7
Thailand	Kanchanaburi	21633	70	29·6	3·69	2·84	0·85
Thailand	Krabi	7368	61	28·1	4·11	3·31	0·81
Thailand	Lamphun	16192	76	28	4·27	3·47	0·8
Thailand	Lampang	37781	51	28·4	3·21	2·16	1·06
Thailand	Lop Buri	29371	59	27·7	3·58	2·93	0·65
Thailand	Maha Sarakham	25121	58	29·4	2·89	2·11	0·78
Thailand	Mukdahan	7688	58	28·1	3·63	2·85	0·78
Thailand	Nan	19042	61	27·9	3·79	3·08	0·71
Thailand	Nakhon Phanom	15974	51	28	3·45	2·57	0·88
Thailand	Nakhon Pathom	25135	61	28·8	3·65	2·78	0·87
Thailand	Nakhon Ratchasima	73701	76	28·8	3·88	3·25	0·63
Thailand	Nakhon Sawan	37873	62	29·2	3·38	2·37	1·01
Thailand	Nakhon Si Thammarat	38612	60	27·8	3·81	3·08	0·73
Thailand	Nong Bua Lam Phu	12259	57	28·9	3·3	2·57	0·72
Thailand	Nong Khai	20118	59	28·8	2·88	2·18	0·7
Thailand	Nonthaburi	30838	64	28·8	3·14	2·26	0·87
Thailand	Narathiwat	15506	52	28·1	2·5	1·81	0·69
Thailand	Pattani	12344	57	28·1	3·49	2·72	0·77
Thailand	Phayao	23941	60	28·3	3·4	2·71	0·69
Thailand	Phichit	15039	54	28·1	2·85	1·78	1·07
Thailand	Phrae	22432	53	28·7	2·82	2·04	0·78
Thailand	Phetchabun	26200	57	27·4	3·3	2·38	0·92
Thailand	Phetchaburi	14351	57	28·2	3·15	2·43	0·72
Thailand	Phitsanulok	31576	54	28·8	3·11	2·16	0·96
Thailand	Prachin Buri	14841	64	28·8	4·1	3·34	0·76
Thailand	Prachuap Khiri Khan	13912	60	28·4	3·7	3·09	0·61
Thailand	Pathum Thani	22274	61	28·2	3·34	2·66	0·68
Thailand	Rayong	18185	65	26·8	3·32	2·56	0·76
Thailand	Roi Et	39160	73	28·9	3·63	3·02	0·61
Thailand	Ratchaburi	29860	74	28·5	4·21	3·46	0·75
Thailand	Sa Kaeo	13335	55	28·1	2·88	2·11	0·77
Thailand	Saraburi	22361	53	28·5	3·16	2·3	0·86

Thailand	Sukhothai	17727	54	28·7	3·13	2·09	1·04
Thailand	Sakon Nakhon	31563	74	28·5	3·81	3·17	0·64
Thailand	Samutprakan	29796	65	29·1	2·89	2·3	0·59
Thailand	Samut Sakhon	15214	55	28·7	3	2·34	0·67
Thailand	Songkhla	33372	58	28·2	3·15	2·4	0·75
Thailand	Suphanburi	24506	67	29·1	3·29	2·5	0·79
Thailand	Surat Thani	21994	79	29·4	4·32	3·7	0·62
Thailand	Si Sa Ket	35365	59	28·3	3·2	2·52	0·68
Thailand	Surin	30862	57	28·4	3·22	2·3	0·93
Thailand	Tak	11373	60	28·4	3·54	2·72	0·82
Thailand	Trang	13314	63	29·2	3·37	2·55	0·82
Thailand	Ubon Ratchathani	48333	74	29·1	3·84	3·21	0·62
Thailand	Udon Thani	44428	75	29·2	3·9	3·18	0·71
Thailand	Uttaradit	19590	56	28·2	3·61	2·71	0·9
Thailand	Yala	10887	58	27·9	3·78	2·99	0·79
Thailand	Yasothon	15133	68	27·7	3·97	3·27	0·69
UK	East	749380	92	18·3	8·84	8·54	0·31
UK	East Midlands	606487	91	17·3	8·59	8·32	0·27
UK	London	845215	92	19·5	9·92	9·47	0·45
UK	North East	407862	90	15·9	7·6	7·27	0·33
UK	North West	1069737	88	16·2	8·52	8·23	0·29
UK	South East	1132649	91	17·9	8·9	8·59	0·31
UK	South West	771244	86	16·3	9·48	9·19	0·29
UK	Wales	472014	89	16·5	9·31	9·08	0·23
UK	West Midlands	771704	92	17·4	8·06	7·85	0·21
UK	Yorkshire & Humber	747424	88	16·2	8·06	7·8	0·26
USA	Akron, OH	107392	87	21·9	7·15	6·76	0·38
USA	Albuquerque, NM	73279	83	24·2	5·99	5·7	0·28
USA	Allentown-Bethlehem, PA	61366	88	23·1	6·86	6·48	0·38
USA	Atlanta, GA	310249	81	25·6	4·9	4·75	0·15
USA	Atlantic City, NJ	49410	85	23·1	7·26	6·79	0·47
USA	Austin, TX	69427	79	28·3	4·06	3·82	0·24
USA	Bakersfield, CA	88852	92	29·2	4·82	4·6	0·22
USA	Baltimore, MD	319591	83	23·9	7·23	6·76	0·47
USA	Barnstable-Yarmouth, MA	51337	92	22·2	6·75	6·58	0·17
USA	Bergen-Passaic, NJ	239023	83	23·9	7·77	7·34	0·43
USA	Birmingham, AL	171109	79	25·6	5·86	5·56	0·31
USA	Boston, MA	475683	86	21·9	8·72	8·32	0·4
USA	Baton Rouge, LA	62561	77	26·9	4·84	4·57	0·27
USA	Brownsville, TX	36059	61	26·9	2·93	2·71	0·22
USA	Buffalo, NY	212201	86	21·1	8·95	8·51	0·44
USA	Canton-Massillon, OH	77288	90	22·8	6·06	5·74	0·31
USA	Charleston, WV	49105	82	22·8	6·48	6·11	0·36
USA	Charlotte, NC	82255	87	26·1	4·85	4·81	0·04
USA	Chattanooga, TN	60219	81	25·3	4·46	4·25	0·22
USA	Chicago, IL	1115158	93	24·7	8·37	8·01	0·37

USA	Cincinnati, OH	171958	83	23·3	6·72	6·3	0·41
USA	Cleveland, OH	404057	86	21·9	7·99	7·56	0·43
USA	Columbia, SC	75994	81	26·4	5·63	5·3	0·33
USA	Columbus, OH	159353	87	23·6	6·05	5·71	0·34
USA	Dallas, TX	260718	79	27·8	4·73	4·34	0·39
USA	Daytona Beach, FL	107272	78	26·9	2·76	2·62	0·14
USA	Dayton, OH	108776	83	22·5	6·8	6·46	0·34
USA	Denver, CO	182600	82	21·1	5·75	5·32	0·43
USA	Des Moines, IA	54488	82	22·5	8·06	7·63	0·43
USA	Detroit, MI	729077	91	23·9	7·26	6·87	0·39
USA	Dutchess County, NY	43055	88	21·9	7·12	6·83	0·29
USA	El Paso, TX	73269	85	27·5	5·09	4·9	0·19
USA	Erie, PA	54723	91	23·1	7·49	7·11	0·37
USA	Flint, MI	75484	91	22·5	7·34	6·89	0·45
USA	Fresno, CA	104033	84	26·9	5·04	4·88	0·16
USA	Ft. Lauderdale, FL	308032	64	26·1	2·17	1·99	0·18
USA	Fort Myers-Cape Coral, FL	88850	44	24·2	2·11	1·62	0·49
USA	Fort Pierce-Port St. Lucie, FL	67004	48	23·9	1·71	1·49	0·22
USA	Fort Worth-Arlington, TX	172892	80	27·8	3·81	3·59	0·21
USA	Galveston, TX	40680	91	28·3	3·7	3·62	0·08
USA	Gary, IN	90669	91	23·9	7·14	6·8	0·34
USA	Grand Rapids, MI	78804	90	22·5	7·76	7·55	0·22
USA	Greensboro, NC	65906	91	26·1	6·3	6·06	0·24
USA	Greenville, SC	58344	81	24·7	5·94	5·69	0·24
USA	Hamilton, OH	49618	84	23·6	6·65	6·35	0·29
USA	Harrisburg-Carlisle, PA	49992	86	23·9	6·59	6·35	0·24
USA	Hartford, CT	159050	84	21·4	7·65	7·03	0·61
USA	Honolulu, HI	75775	99	29·2	2·87	2·87	0
USA	Houston, TX	366340	63	25·3	3·38	3·23	0·15
USA	Indianapolis, IN	149459	86	23·6	6·01	5·75	0·27
USA	Jacksonville, FL	124017	77	26·7	4·54	4·28	0·26
USA	Jersey City, NJ	103084	84	24·2	7·31	6·93	0·39
USA	Kansas City, MO-KS	218933	82	23·9	6·86	6·43	0·43
USA	Knoxville, TN	80418	81	24·4	5·42	5·05	0·36
USA	Lakeland-Winter Haven, FL	95395	61	26·1	2·99	2·74	0·25
USA	Lancaster, PA	80724	91	24·4	6·3	6·09	0·21
USA	Lansing, MI	37393	90	22·2	8·06	7·64	0·42
USA	Las Vegas, NV-AZ	182220	78	30	4·63	4·24	0·39
USA	Los Angeles, CA	1239036	95	22·8	2·48	2·34	0·14
USA	Louisville, KY	139347	80	24·2	6·54	6·07	0·47
USA	Little Rock, AR	63901	79	26·1	5·15	4·8	0·36
USA	Lubbock, TX	35407	83	26·1	5·69	5·51	0·18
USA	Madison, WI	48763	90	22·5	7·24	6·96	0·28
USA	McAllen-Edinburg-Mission, TX	49998	71	29·2	3·19	2·98	0·21
USA	Melbourne-Titusville-Palm Bay, FL	88449	73	26·7	3·44	3·28	0·16
USA	Memphis, TN	152003	79	26·4	6·54	6·28	0·26

USA	Miami, FL	372130	39	24·7	1·9	1·07	0·83
USA	Middlesex, NJ	110324	90	24·2	8·01	7·57	0·44
USA	Milwaukee, WI	232056	83	20·8	9·7	9·14	0·56
USA	Minneapolis-St. Paul, MN	241475	87	22·2	8·21	7·83	0·37
USA	Mobile, AL	72746	78	26·7	3·34	3·24	0·11
USA	Monmouth-Ocean, NJ	235036	90	24·2	6·94	6·69	0·25
USA	Myrtle Beach, SC	30268	82	25·8	4·9	4·73	0·17
USA	Naples, FL	36951	49	24·7	2·28	1·91	0·37
USA	Nashua, NH	51115	91	22·2	8·37	7·97	0·41
USA	Nashville, TN	97358	80	25	6·81	6·36	0·45
USA	Nassau-Suffolk, NY	460192	85	22·2	8·89	8·57	0·33
USA	Newark, NJ	220980	83	23·9	6·76	6·22	0·54
USA	Newburgh, NY	49890	91	23·3	7·59	7·32	0·27
USA	New Haven-Meriden, CT	157415	85	21·7	8·57	7·98	0·59
USA	New London, CT	40419	91	23·3	7·2	6·84	0·36
USA	New York, NY	1367085	80	23·1	7·71	6·9	0·82
USA	Oakland, CA	325028	95	20·8	4·09	3·95	0·14
USA	Ocala, FL	58345	73	26·7	3·49	3·36	0·13
USA	Oklahoma City, OK	118753	90	28·1	5·36	5·2	0·16
USA	Omaha, NE	71558	84	23·3	7·6	7·02	0·58
USA	Orange County, CA	320343	96	25·6	2·04	1·99	0·05
USA	Orlando, FL	157019	73	27·2	2·6	2·41	0·19
USA	Pensacola, FL	50546	77	26·9	4·84	4·53	0·31
USA	Philadelphia, PA-NJ	911888	80	23·3	8·17	7·71	0·47
USA	Phoenix, AZ	386802	65	29·2	2·92	2·74	0·18
USA	Pittsburgh, PA	317935	90	23·3	6·68	6·31	0·37
USA	Portland, ME	46217	90	20·6	8·08	7·67	0·42
USA	Portland, OR	210301	91	21·4	6·86	6·49	0·37
USA	Providence-Fall River, RI-MA	36108	91	23·3	7·23	7	0·22
USA	Punta Gorda, FL	37773	59	25·8	2·62	2·46	0·16
USA	Raleigh, NC	58561	84	25·3	5·41	5·14	0·27
USA	Reading, PA	72337	80	24·4	5·37	4·94	0·43
USA	Riverside-San Bernardino, CA	433285	95	28·3	2·78	2·74	0·04
USA	Rochester, NY	127040	89	21·7	7·71	7·28	0·43
USA	Rockford, IL	46380	85	21·9	8·02	7·65	0·38
USA	Sacramento, CA	172136	92	25·3	4·23	4·07	0·16
USA	Saginaw, MI	39515	87	21·4	8·05	7·61	0·43
USA	Salinas, CA	45929	97	20·6	3·95	3·87	0·08
USA	Salt Lake City, UT	89770	86	24·7	6·32	6·03	0·29
USA	San Antonio, TX	186461	89	30	3·01	2·9	0·11
USA	Sarasota-Bradenton, FL	151551	47	23·9	1·85	1·51	0·34
USA	Scranton--Wilkes-Barre--Hazleton, PA	150119	84	20·8	7·71	7·39	0·32
USA	San Diego, CA	369956	96	23·3	3·45	3·39	0·06
USA	Seattle, WA	225451	92	19·4	6·67	6·39	0·28
USA	San Francisco, CA	248607	97	20·8	3·33	3·27	0·06
USA	Shreveport, LA	51716	78	26·9	5·33	4·97	0·36

USA	San Jose, CA	176066	94	23·3	3·14	2·97	0·17
USA	Spokane, WA	68681	89	20·8	7·33	6·92	0·41
USA	Springfield, MA	94971	84	21·4	8·65	8·08	0·58
USA	Stamford-Norwalk, CT	142216	86	22·5	7·56	7·2	0·36
USA	St. Louis, MO-IL	312923	91	27·2	5·87	5·62	0·25
USA	Stockton-Lodi, CA	82225	90	25·6	6·02	5·86	0·16
USA	Syracuse, NY	84451	86	21·1	7·36	6·87	0·49
USA	Tacoma, WA	96086	93	20	6·26	6·17	0·1
USA	Tampa-St. Petersburg-Clearwater, FL	158555	54	25	2·69	2·44	0·25
USA	Toledo, OH	92004	84	21·7	7·91	7·42	0·5
USA	Trenton, NJ	58430	90	24·2	6·9	6·63	0·27
USA	Tucson, AZ	131053	78	28·9	4·27	4·04	0·24
USA	Tulsa, OK	95475	84	26·9	5·74	5·63	0·11
USA	Utica-Rome, NY	53724	90	21·7	7·59	7·26	0·33
USA	Ventura County, CA	87603	96	21·7	2·77	2·69	0·08
USA	Virginia Beach, VA	187233	83	25·3	5·26	4·94	0·32
USA	Washington, DC-MD-VA	141028	87	25·8	6·44	6·07	0·37
USA	Wichita, KS	68542	81	25	6	5·55	0·45
USA	Wilmington, DE	76254	84	23·6	7·11	6·63	0·48
USA	Worcester, MA	135785	85	20	8·23	7·68	0·55
USA	West Palm Beach-Boca Raton, FL	233887	40	24·2	1·99	1·28	0·71
USA	York, PA	62767	82	22·5	6·92	6·54	0·38
USA	Youngstown-Warren, OH	86656	85	20·8	6·69	6·36	0·33

Additional figures

***Figure S1*: Overall cumulative exposure-response relationships in 384 locations**

These exposure-response relationships are computed as BLUP (with 95%eCI) in the 384 locations of the 13 countries, with related temperature distributions. The minimum mortality temperature (MMT) and the 2.5^{th} and 97.5^{th} percentiles are added as dotted and dashed lines, respectively.

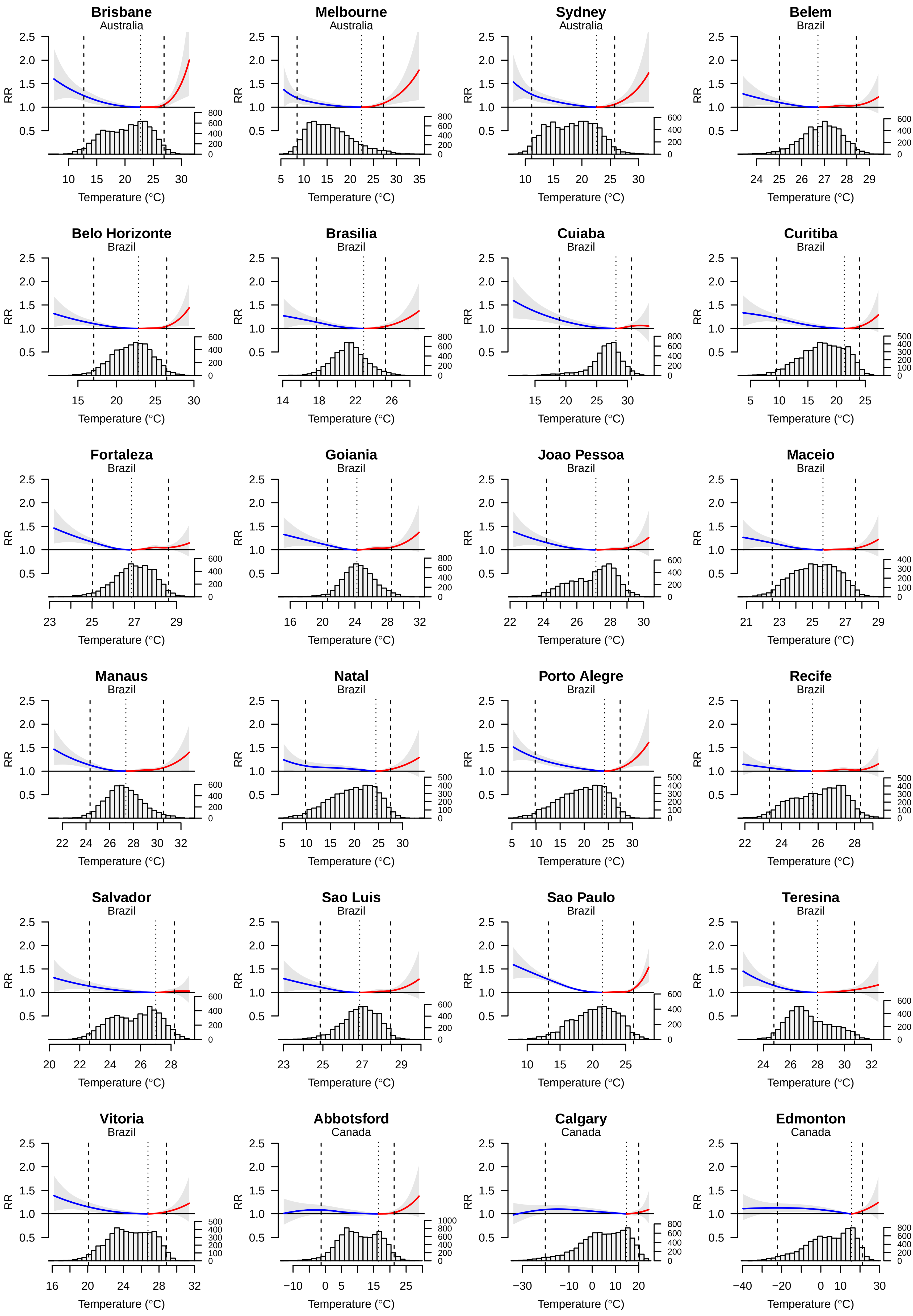
Brisbane
Australia
Melbourne
Australia
Sydney
Australia
Belem
Brazil
Belo Horizonte
Brazil
Brasilia
Brazil
Cuiaba
Brazil
Curitiba
Brazil
Fortaleza
Brazil
Goiania
Brazil
Joao Pessoa
Brazil
Maceio
Brazil
Manaus
Brazil
Natal
Brazil
Porto Alegre
Brazil
Recife
Brazil
Salvador
Brazil
Sao Luis
Brazil
Sao Paulo
Brazil
Teresina
Brazil
Vitoria
Brazil
Abbotsford
Canada
Calgary
Canada
Edmonton
Canada
RR
Temperature (°C)

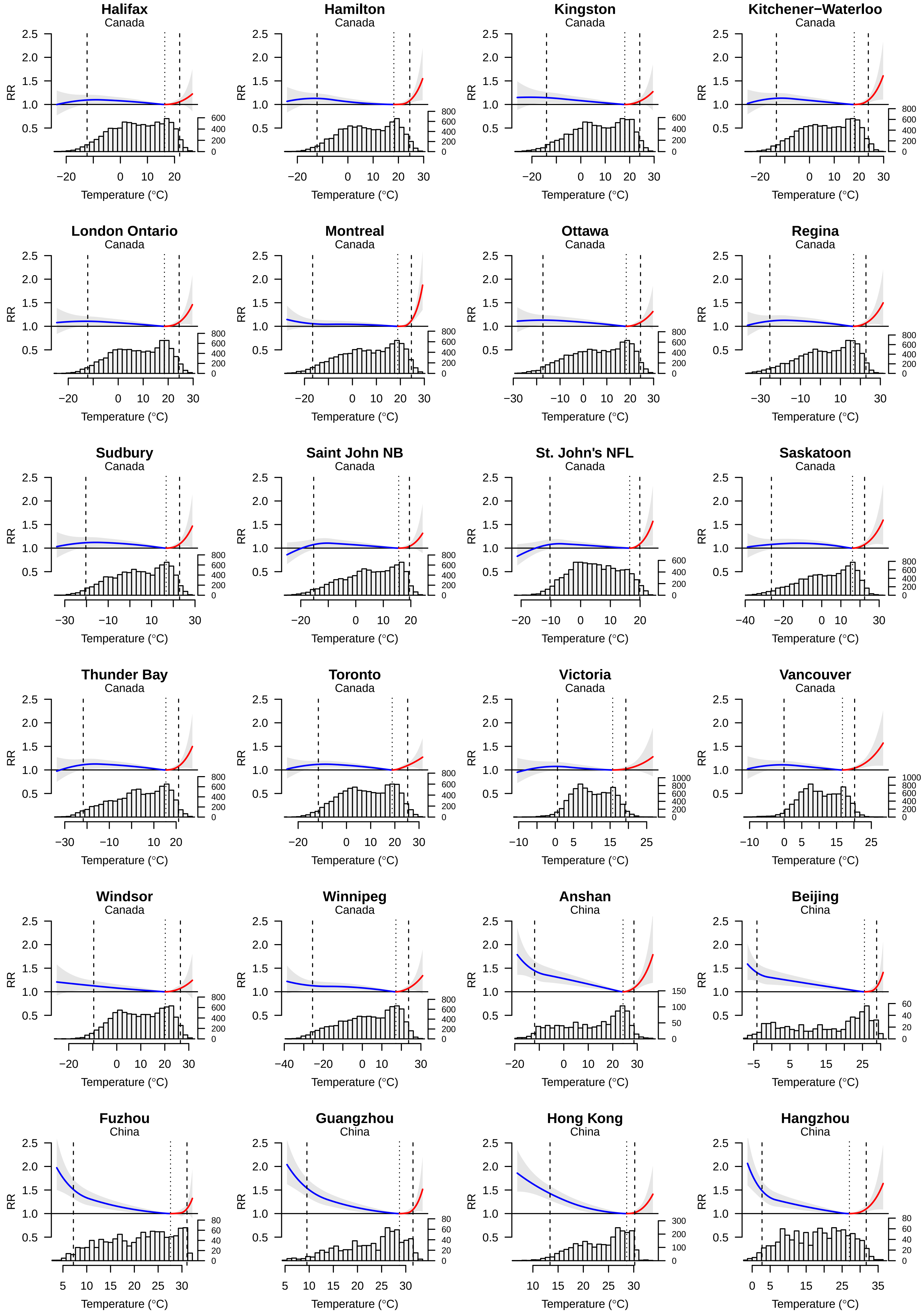

Halifax
Canada
Hamilton
Canada
Kingston
Canada
Kitchener−Waterloo
Canada
London Ontario
Canada
Montreal
Canada
Ottawa
Canada
Regina
Canada
Sudbury
Canada
Saint John NB
Canada
St. John's NFL
Canada
Saskatoon
Canada
Thunder Bay
Canada
Toronto
Canada
Victoria
Canada
Vancouver
Canada
Windsor
Canada
Winnipeg
Canada
Anshan
China
Beijing
China
Fuzhou
China
Guangzhou
China
Hong Kong
China
Hangzhou
China
RR
Temperature (°C)

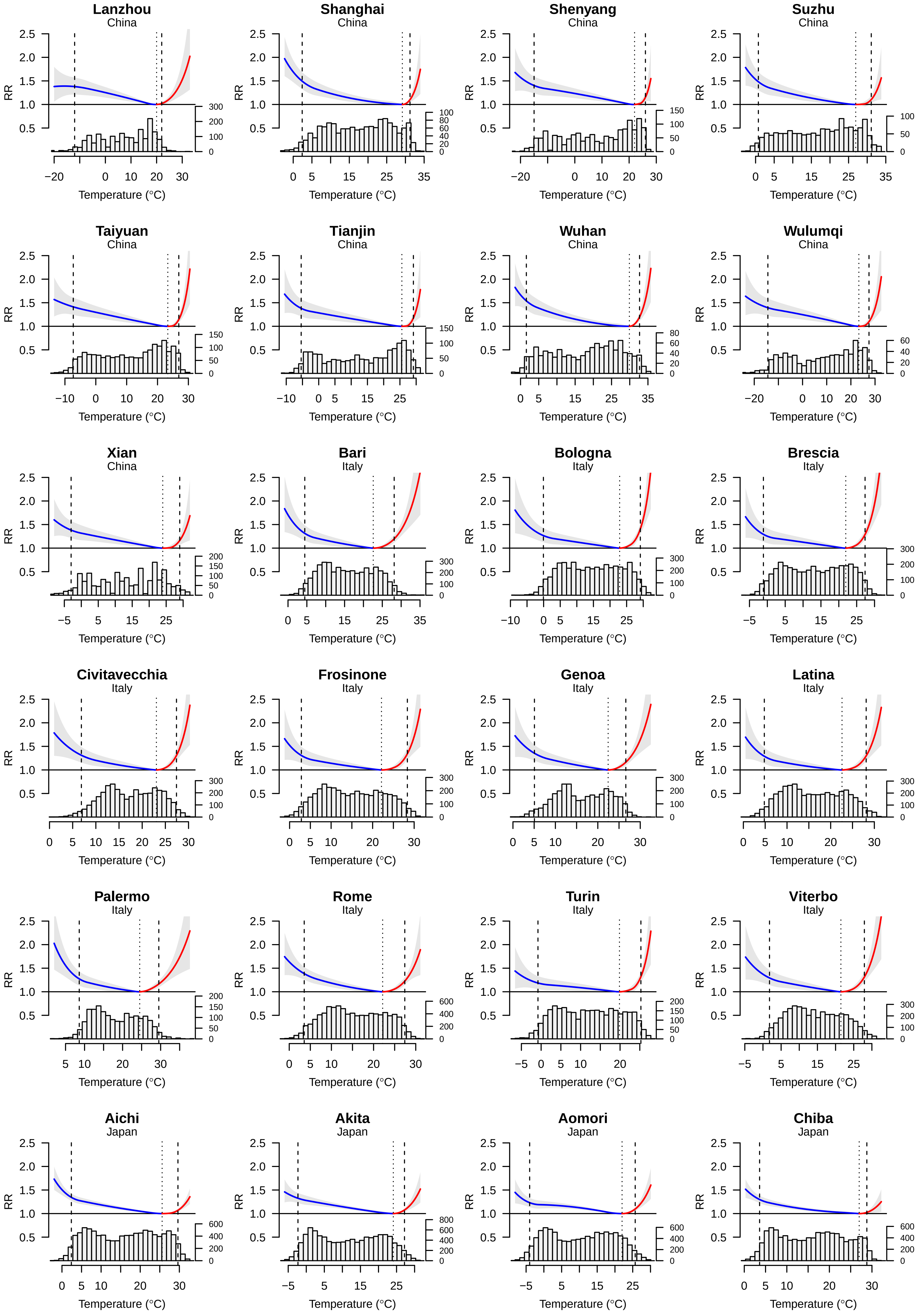
Lanzhou
China
Shanghai
China
Shenyang
China
Suzhou
China
Taiyuan
China
Tianjin
China
Wuhan
China
Wulumqi
China
Xian
China
Bari
Italy
Bologna
Italy
Brescia
Italy
Civitavecchia
Italy
Frosinone
Italy
Genoa
Italy
Latina
Italy
Palermo
Italy
Rome
Italy
Turin
Italy
Viterbo
Italy
Aichi
Japan
Akita
Japan
Aomori
Japan
Chiba
Japan
RR
Temperature (°C)

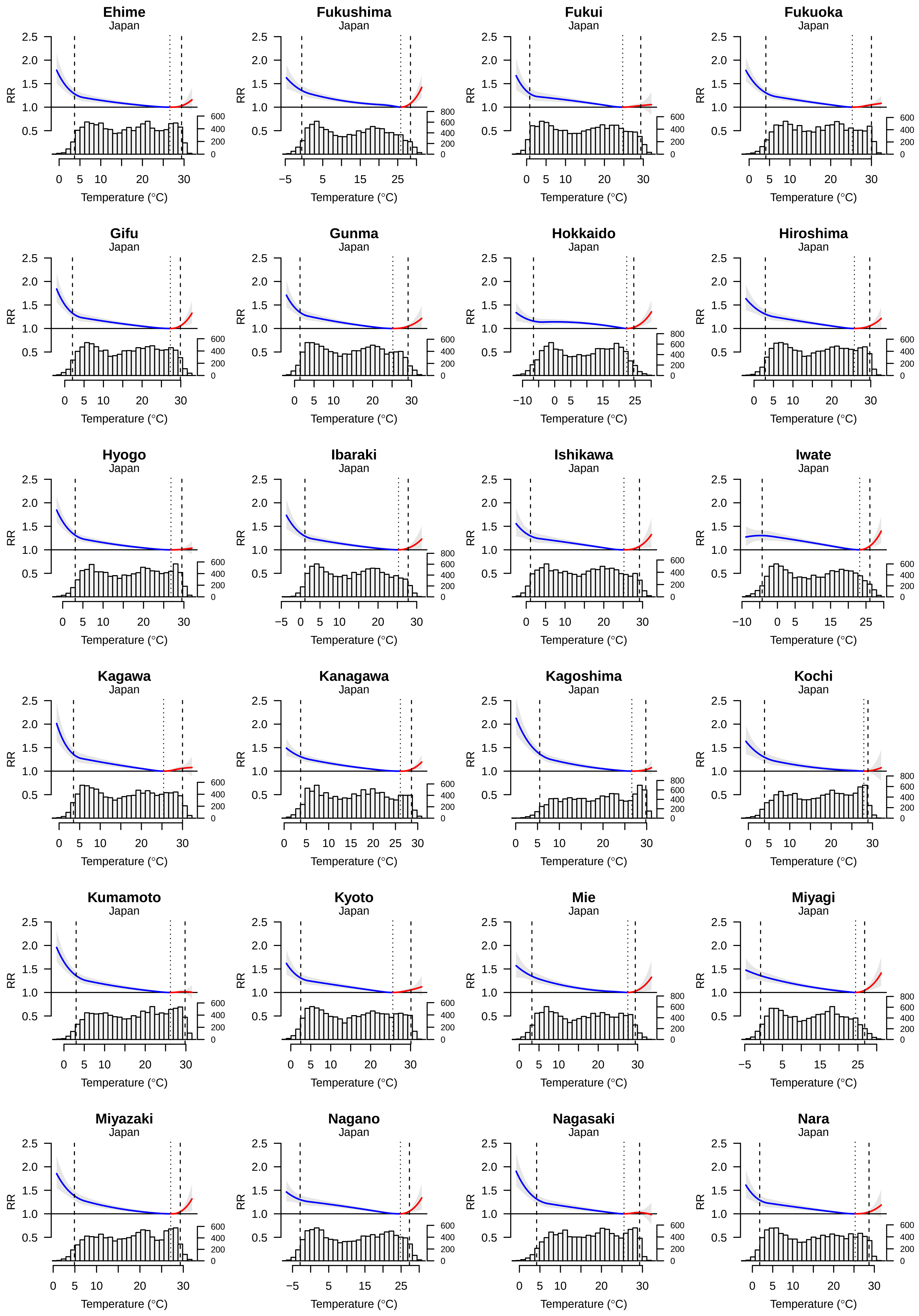

Ehime
Japan
Fukushima
Japan
Fukui
Japan
Fukuoka
Japan
Gifu
Japan
Gunma
Japan
Hokkaido
Japan
Hiroshima
Japan
Hyogo
Japan
Ibaraki
Japan
Ishikawa
Japan
Iwate
Japan
Kagawa
Japan
Kanagawa
Japan
Kagoshima
Japan
Kochi
Japan
Kumamoto
Japan
Kyoto
Japan
Mie
Japan
Miyagi
Japan
Miyazaki
Japan
Nagano
Japan
Nagasaki
Japan
Nara
Japan
RR
Temperature (°C)

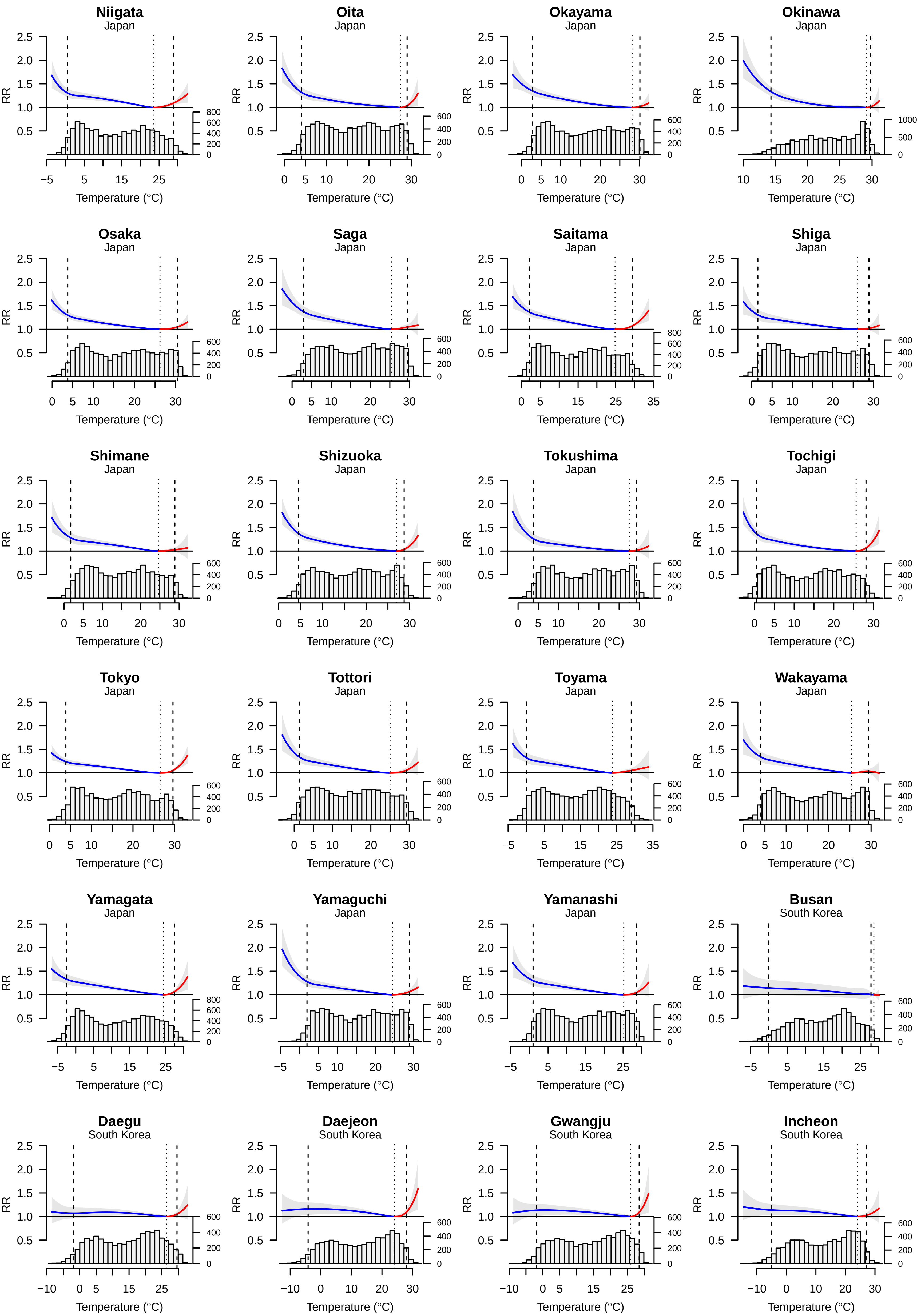

Niigata
Japan
Oita
Japan
Okayama
Japan
Okinawa
Japan
Osaka
Japan
Saga
Japan
Saitama
Japan
Shiga
Japan
Shimane
Japan
Shizuoka
Japan
Tokushima
Japan
Tochigi
Japan
Tokyo
Japan
Tottori
Japan
Toyama
Japan
Wakayama
Japan
Yamagata
Japan
Yamaguchi
Japan
Yamanashi
Japan
Busan
South Korea
Daegu
South Korea
Daejeon
South Korea
Gwangju
South Korea
Incheon
South Korea
RR
Temperature (°C)

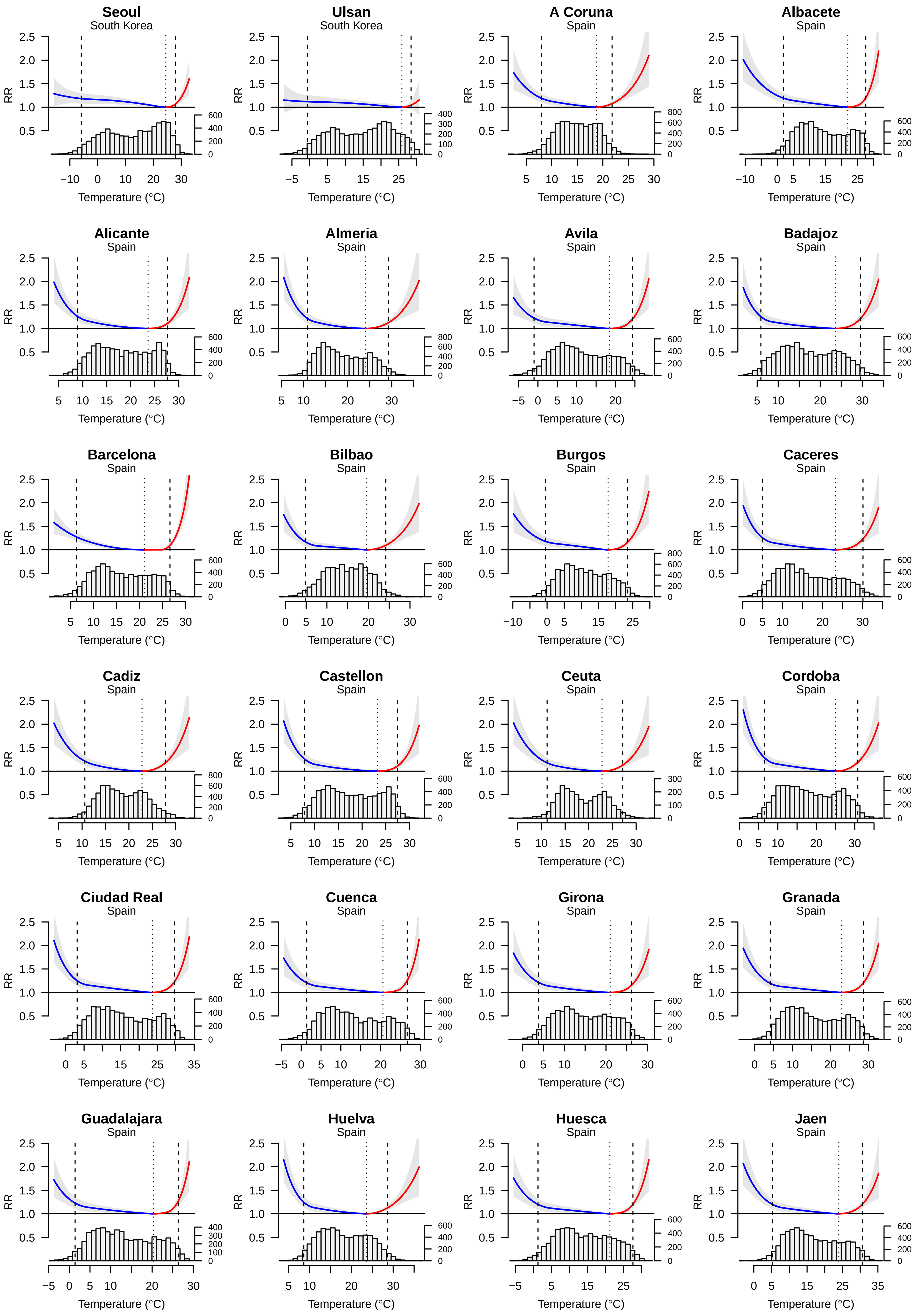
Seoul
South Korea
Ulsan
South Korea
A Coruna
Spain
Albacete
Spain
Alicante
Spain
Almeria
Spain
Avila
Spain
Badajoz
Spain
Barcelona
Spain
Bilbao
Spain
Burgos
Spain
Caceres
Spain
Cadiz
Spain
Castellon
Spain
Ceuta
Spain
Cordoba
Spain
Ciudad Real
Spain
Cuenca
Spain
Girona
Spain
Granada
Spain
Guadalajara
Spain
Huelva
Spain
Huesca
Spain
Jaen
Spain
RR
Temperature (°C)

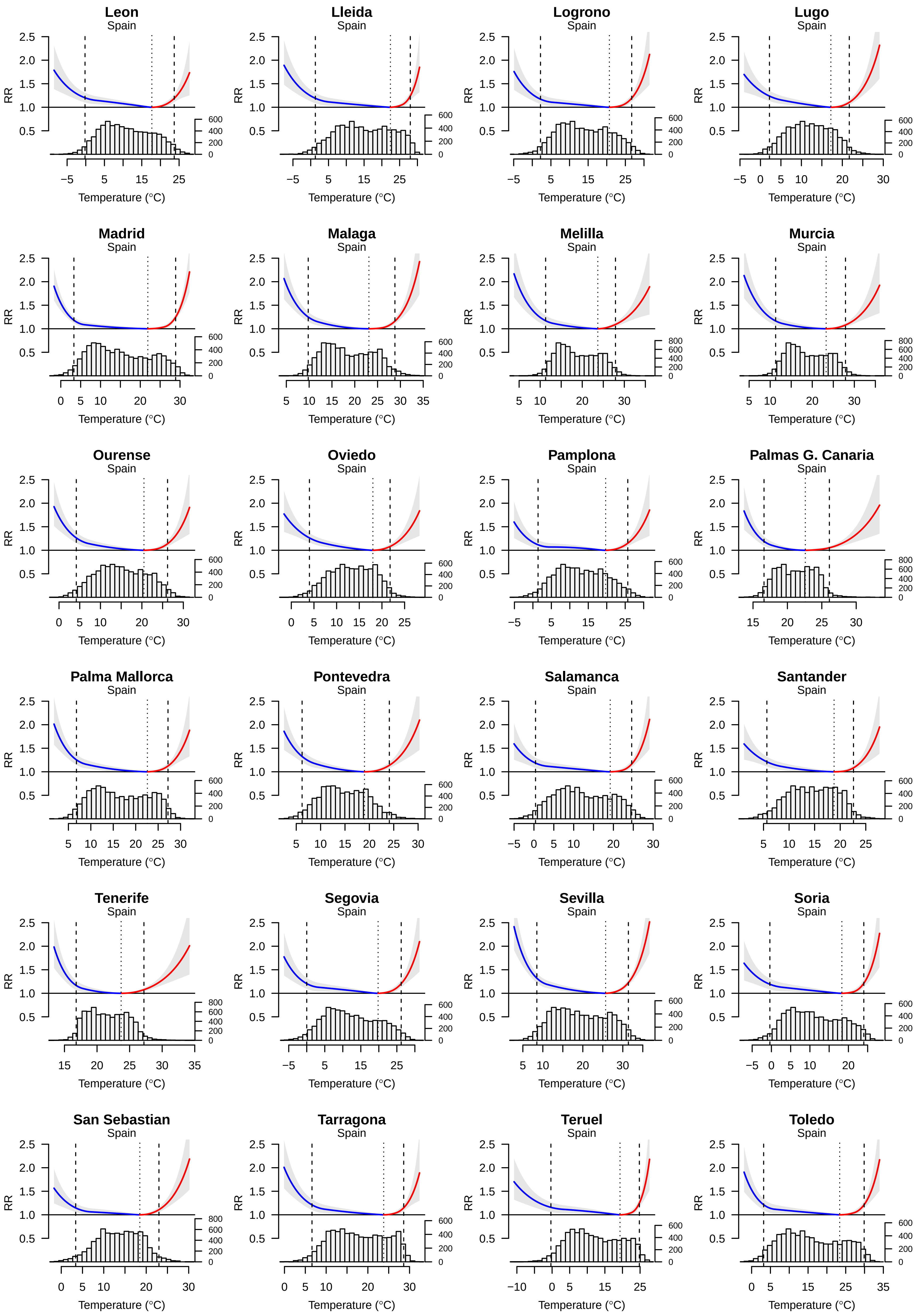

Leon
Spain
Lleida
Spain
Logrono
Spain
Lugo
Spain
Madrid
Spain
Malaga
Spain
Melilla
Spain
Murcia
Spain
Ourense
Spain
Oviedo
Spain
Pamplona
Spain
Palmas G. Canaria
Spain
Palma Mallorca
Spain
Pontevedra
Spain
Salamanca
Spain
Santander
Spain
Tenerife
Spain
Segovia
Spain
Sevilla
Spain
Soria
Spain
San Sebastian
Spain
Tarragona
Spain
Teruel
Spain
Toledo
Spain
RR
Temperature (°C)

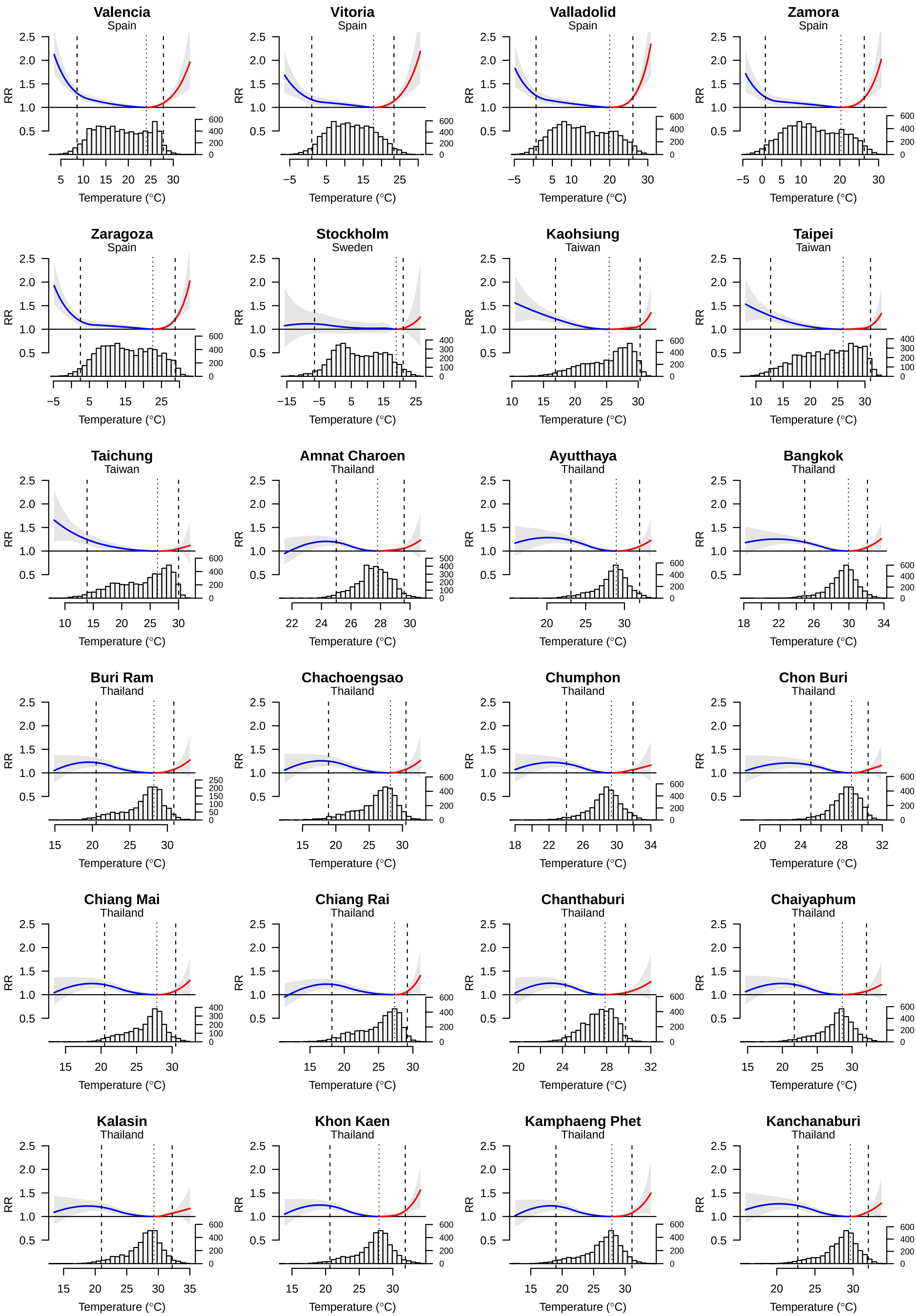
Valencia
Spain
Vitoria
Spain
Valladolid
Spain
Zamora
Spain
Zaragoza
Spain
Stockholm
Sweden
Kaohsiung
Taiwan
Taipei
Taiwan
Taichung
Taiwan
Amnat Charoen
Thailand
Ayutthaya
Thailand
Bangkok
Thailand
Buri Ram
Thailand
Chachoengsao
Thailand
Chumphon
Thailand
Chon Buri
Thailand
Chiang Mai
Thailand
Chiang Rai
Thailand
Chanthaburi
Thailand
Chaiyaphum
Thailand
Kalasin
Thailand
Khon Kaen
Thailand
Kamphaeng Phet
Thailand
Kanchanaburi
Thailand
RR
Temperature (°C)

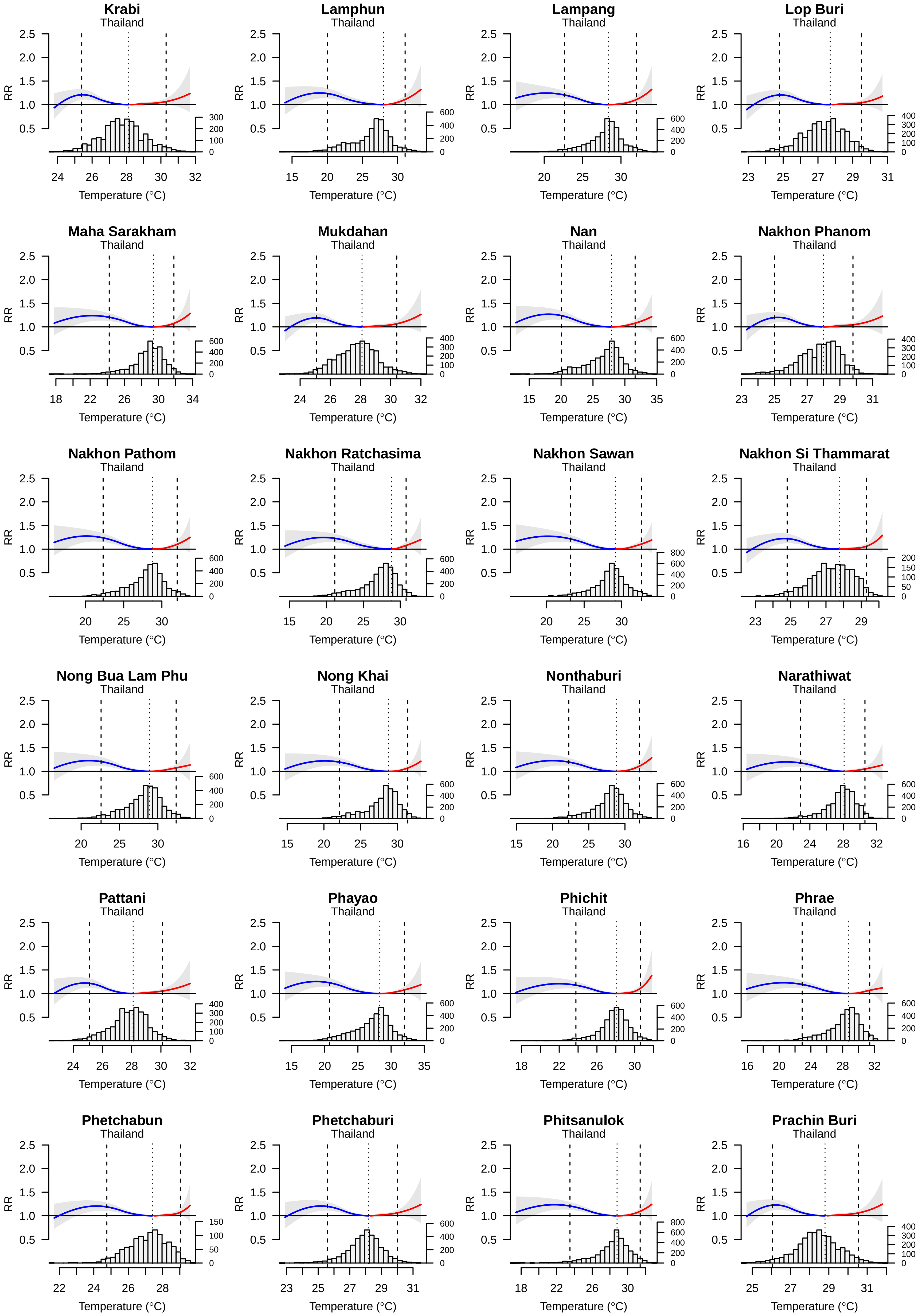
Krabi
Thailand
Lamphun
Thailand
Lampang
Thailand
Lop Buri
Thailand
Maha Sarakham
Thailand
Mukdahan
Thailand
Nan
Thailand
Nakhon Phanom
Thailand
Nakhon Pathom
Thailand
Nakhon Ratchasima
Thailand
Nakhon Sawan
Thailand
Nakhon Si Thammarat
Thailand
Nong Bua Lam Phu
Thailand
Nong Khai
Thailand
Nonthaburi
Thailand
Narathiwat
Thailand
Pattani
Thailand
Phayao
Thailand
Phichit
Thailand
Phrae
Thailand
Phetchabun
Thailand
Phetchaburi
Thailand
Phitsanulok
Thailand
Prachin Buri
Thailand
RR
Temperature (°C)

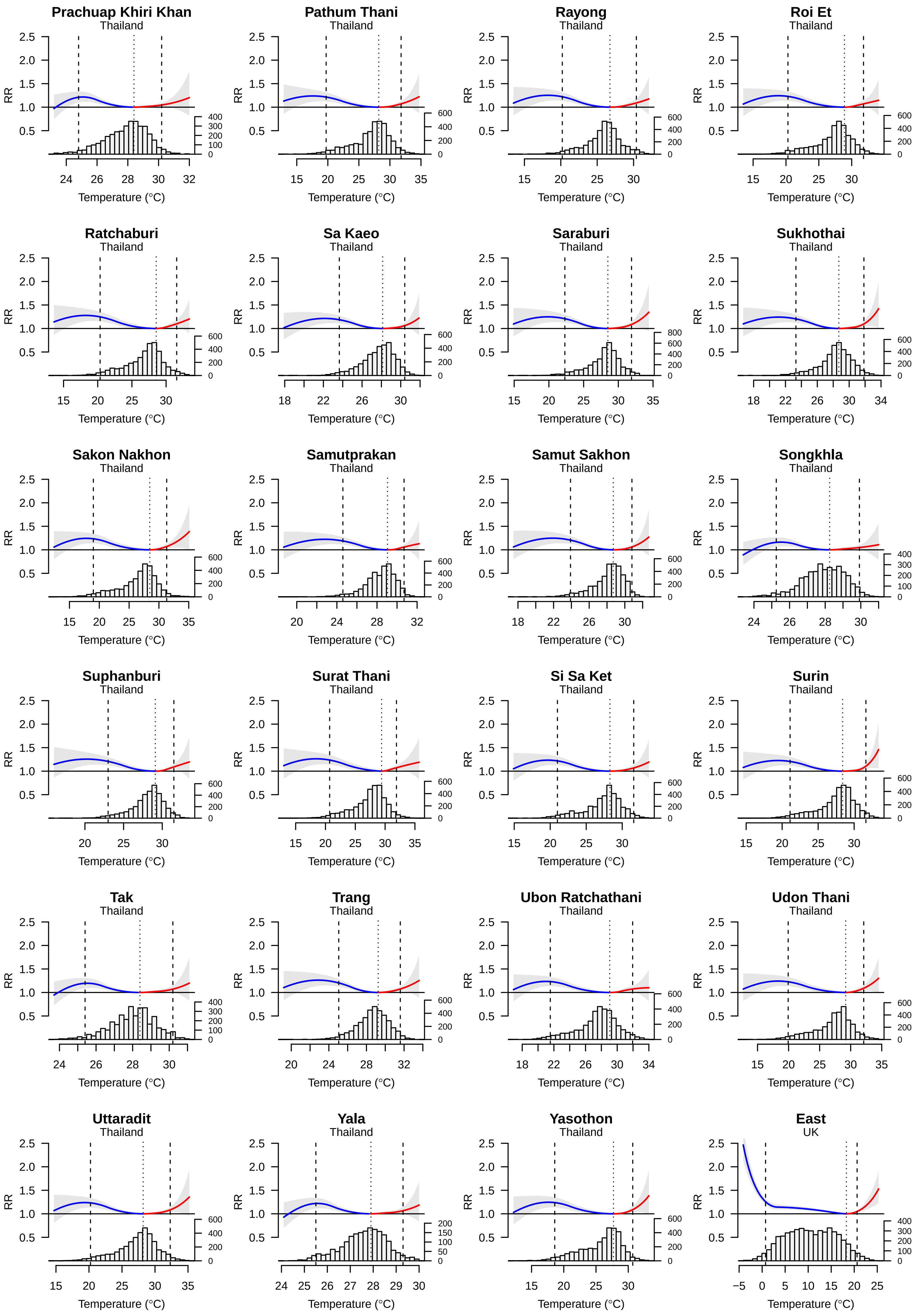
Prachuap Khiri Khan
Thailand
Pathum Thani
Thailand
Rayong
Thailand
Roi Et
Thailand
Ratchaburi
Thailand
Sa Kaeo
Thailand
Saraburi
Thailand
Sukhothai
Thailand
Sakon Nakhon
Thailand
Samutprakan
Thailand
Samut Sakhon
Thailand
Songkhla
Thailand
Suphanburi
Thailand
Surat Thani
Thailand
Si Sa Ket
Thailand
Surin
Thailand
Tak
Thailand
Trang
Thailand
Ubon Ratchathani
Thailand
Udon Thani
Thailand
Uttaradit
Thailand
Yala
Thailand
Yasothon
Thailand
East
UK
RR
Temperature (°C)

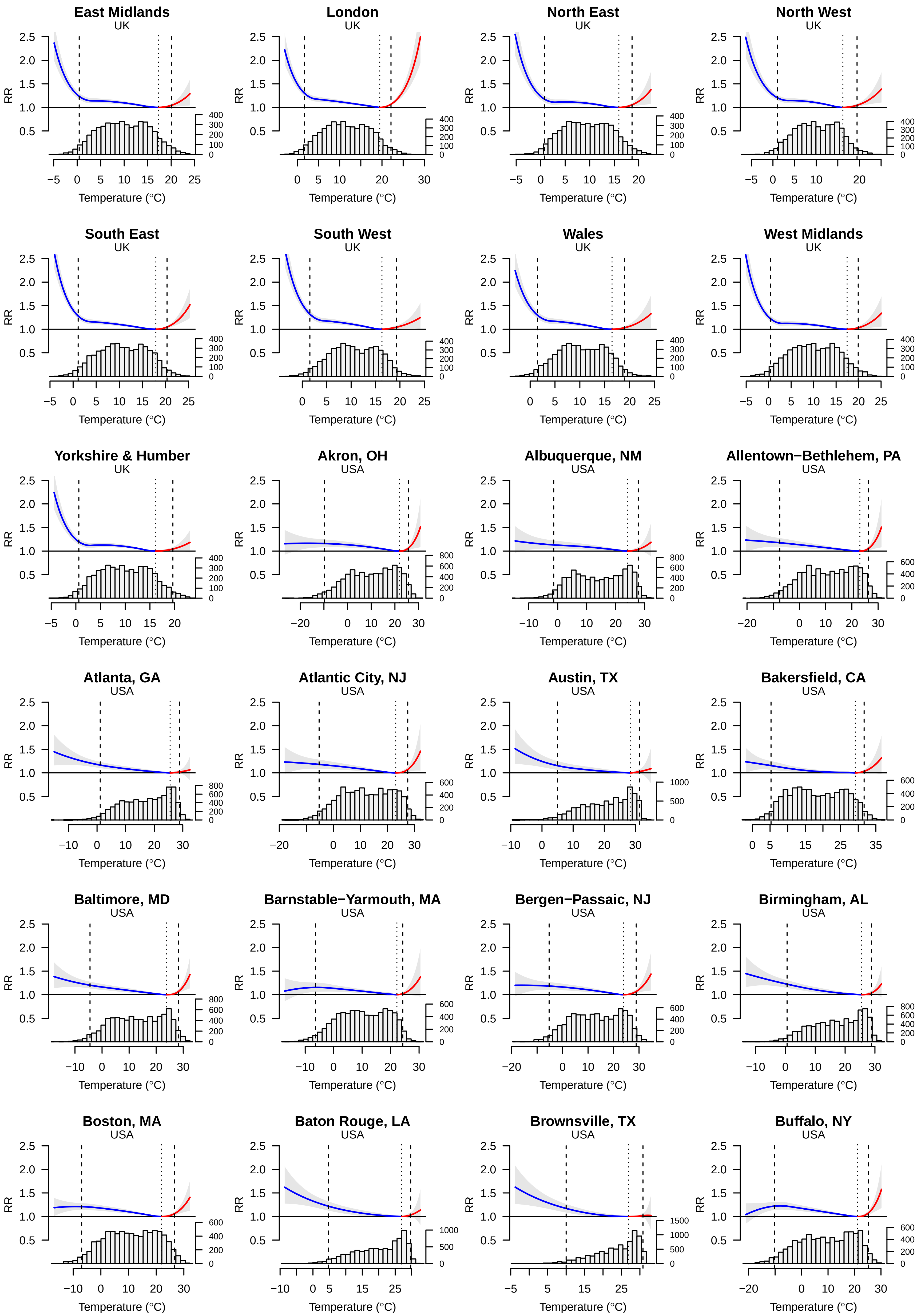
East Midlands
UK
London
UK
North East
UK
North West
UK
South East
UK
South West
UK
Wales
UK
West Midlands
UK
Yorkshire & Humber
UK
Akron, OH
USA
Albuquerque, NM
USA
Allentown−Bethlehem, PA
USA
Atlanta, GA
USA
Atlantic City, NJ
USA
Austin, TX
USA
Bakersfield, CA
USA
Baltimore, MD
USA
Barnstable−Yarmouth, MA
USA
Bergen−Passaic, NJ
USA
Birmingham, AL
USA
Boston, MA
USA
Baton Rouge, LA
USA
Brownsville, TX
USA
Buffalo, NY
USA
RR
Temperature (°C)

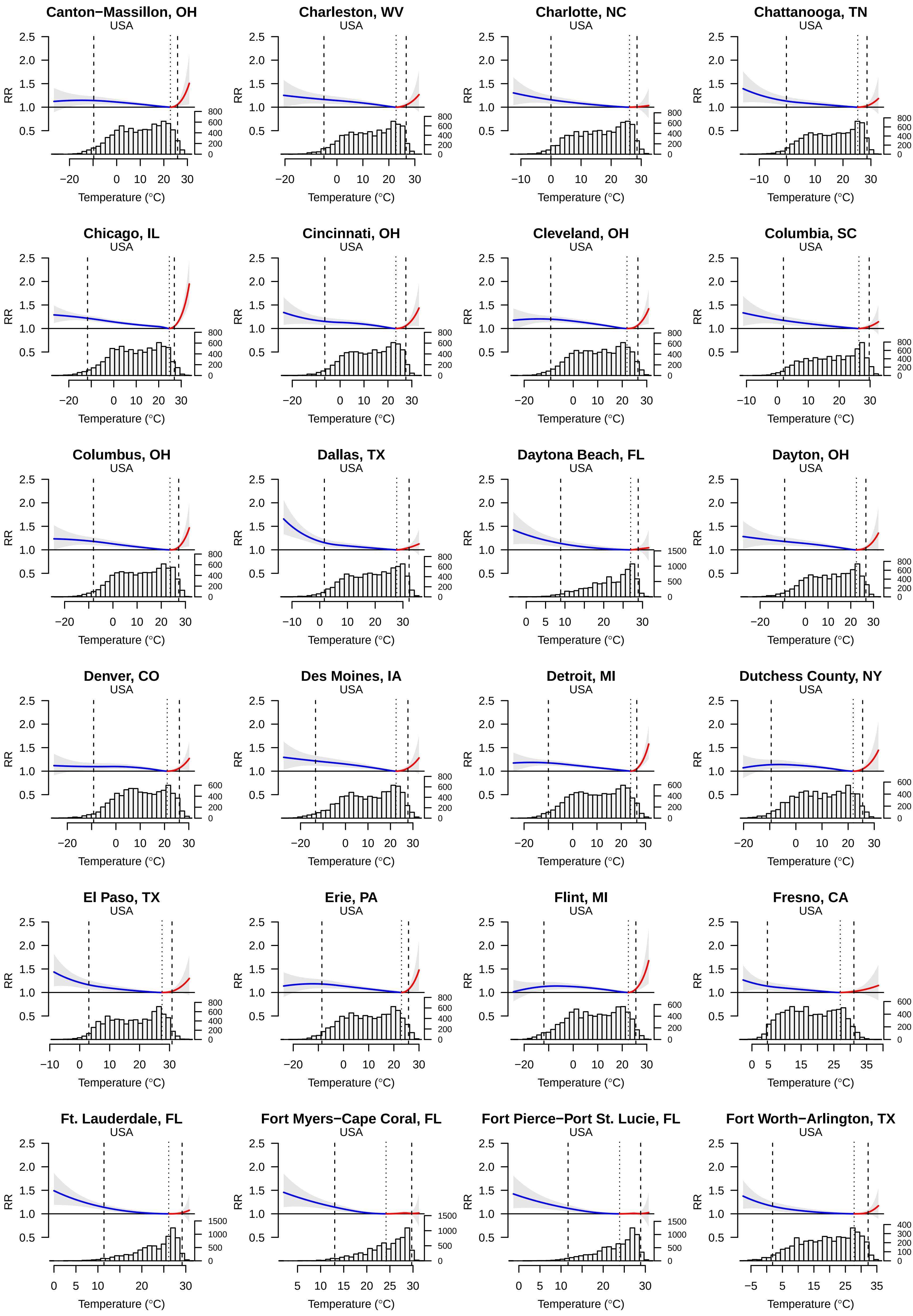

Canton−Massillon, OH
USA
Charleston, WV
USA
Charlotte, NC
USA
Chattanooga, TN
USA
Chicago, IL
USA
Cincinnati, OH
USA
Cleveland, OH
USA
Columbia, SC
USA
Columbus, OH
USA
Dallas, TX
USA
Daytona Beach, FL
USA
Dayton, OH
USA
Denver, CO
USA
Des Moines, IA
USA
Detroit, MI
USA
Dutchess County, NY
USA
El Paso, TX
USA
Erie, PA
USA
Flint, MI
USA
Fresno, CA
USA
Ft. Lauderdale, FL
USA
Fort Myers−Cape Coral, FL
USA
Fort Pierce−Port St. Lucie, FL
USA
Fort Worth−Arlington, TX
USA
RR
Temperature (°C)

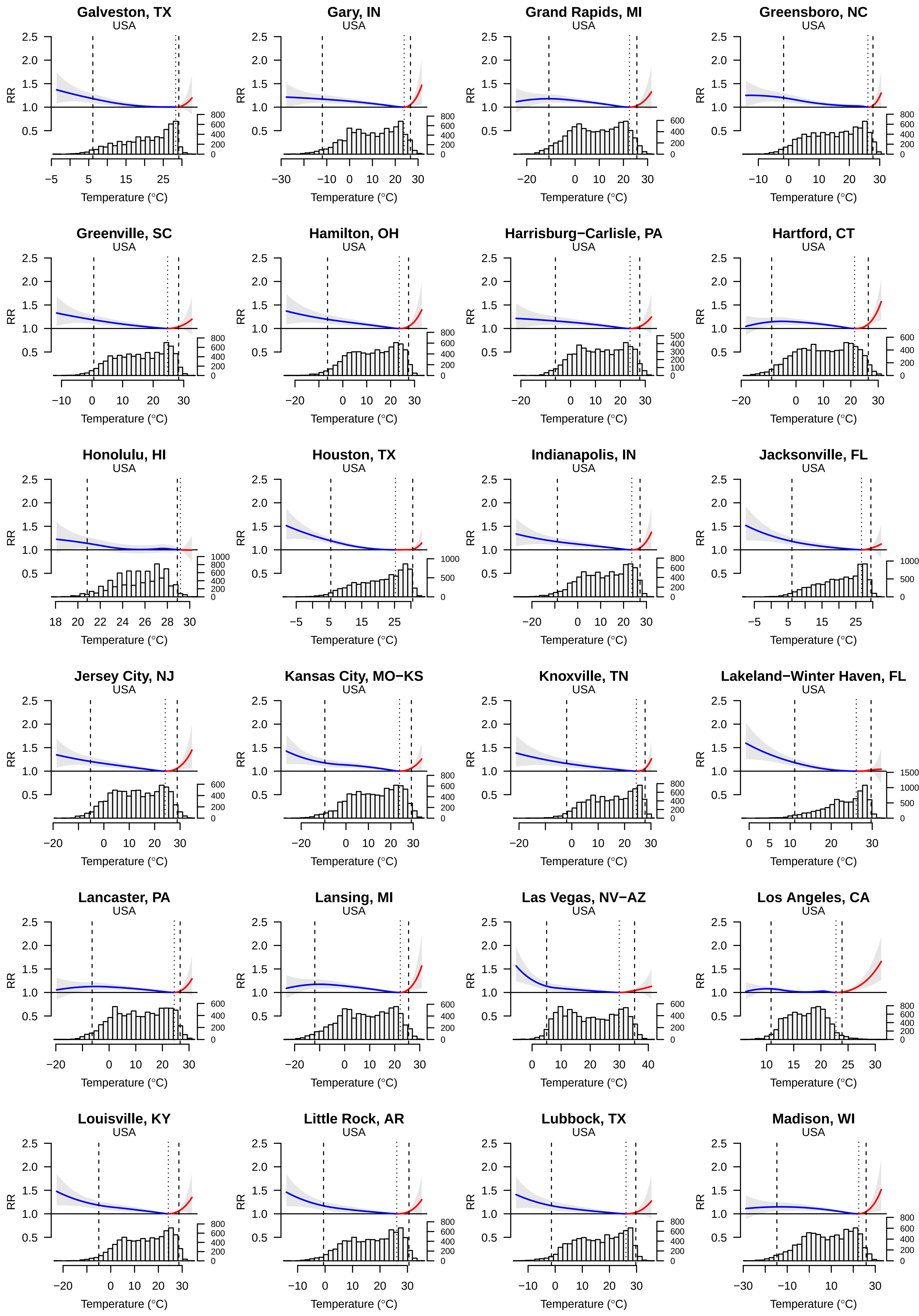
Galveston, TX
USA
Gary, IN
USA
Grand Rapids, MI
USA
Greensboro, NC
USA
Greenville, SC
USA
Hamilton, OH
USA
Harrisburg−Carlisle, PA
USA
Hartford, CT
USA
Honolulu, HI
USA
Houston, TX
USA
Indianapolis, IN
USA
Jacksonville, FL
USA
Jersey City, NJ
USA
Kansas City, MO−KS
USA
Knoxville, TN
USA
Lakeland−Winter Haven, FL
USA
Lancaster, PA
USA
Lansing, MI
USA
Las Vegas, NV−AZ
USA
Los Angeles, CA
USA
Louisville, KY
USA
Little Rock, AR
USA
Lubbock, TX
USA
Madison, WI
USA
RR
Temperature (°C)

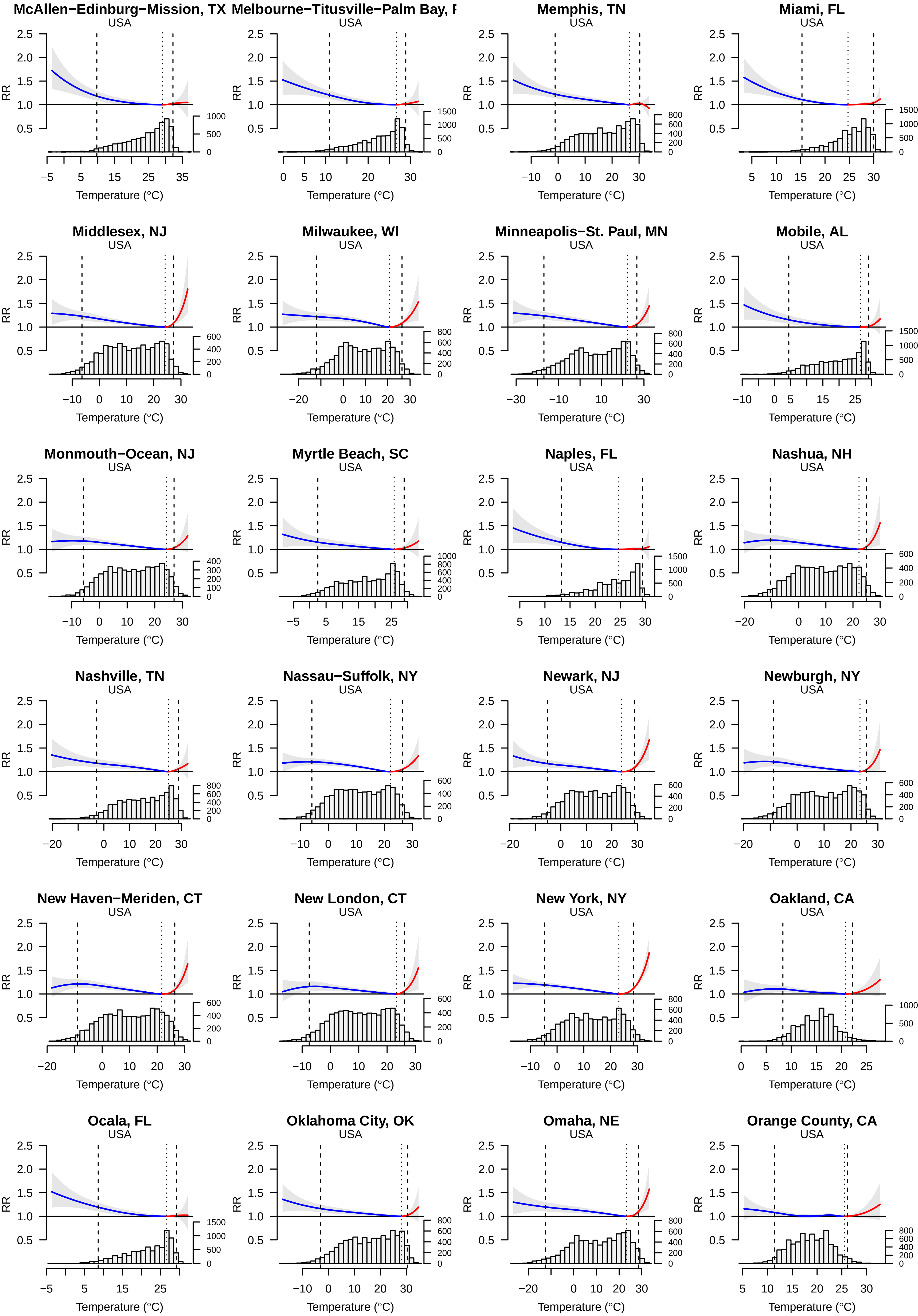
McAllen−Edinburg−Mission, TX
USA
Melbourne−Titusville−Palm Bay, F
USA
Memphis, TN
USA
Miami, FL
USA
Middlesex, NJ
USA
Milwaukee, WI
USA
Minneapolis−St. Paul, MN
USA
Mobile, AL
USA
Monmouth−Ocean, NJ
USA
Myrtle Beach, SC
USA
Naples, FL
USA
Nashua, NH
USA
Nashville, TN
USA
Nassau−Suffolk, NY
USA
Newark, NJ
USA
Newburgh, NY
USA
New Haven−Meriden, CT
USA
New London, CT
USA
New York, NY
USA
Oakland, CA
USA
Ocala, FL
USA
Oklahoma City, OK
USA
Omaha, NE
USA
Orange County, CA
USA
RR
Temperature (°C)

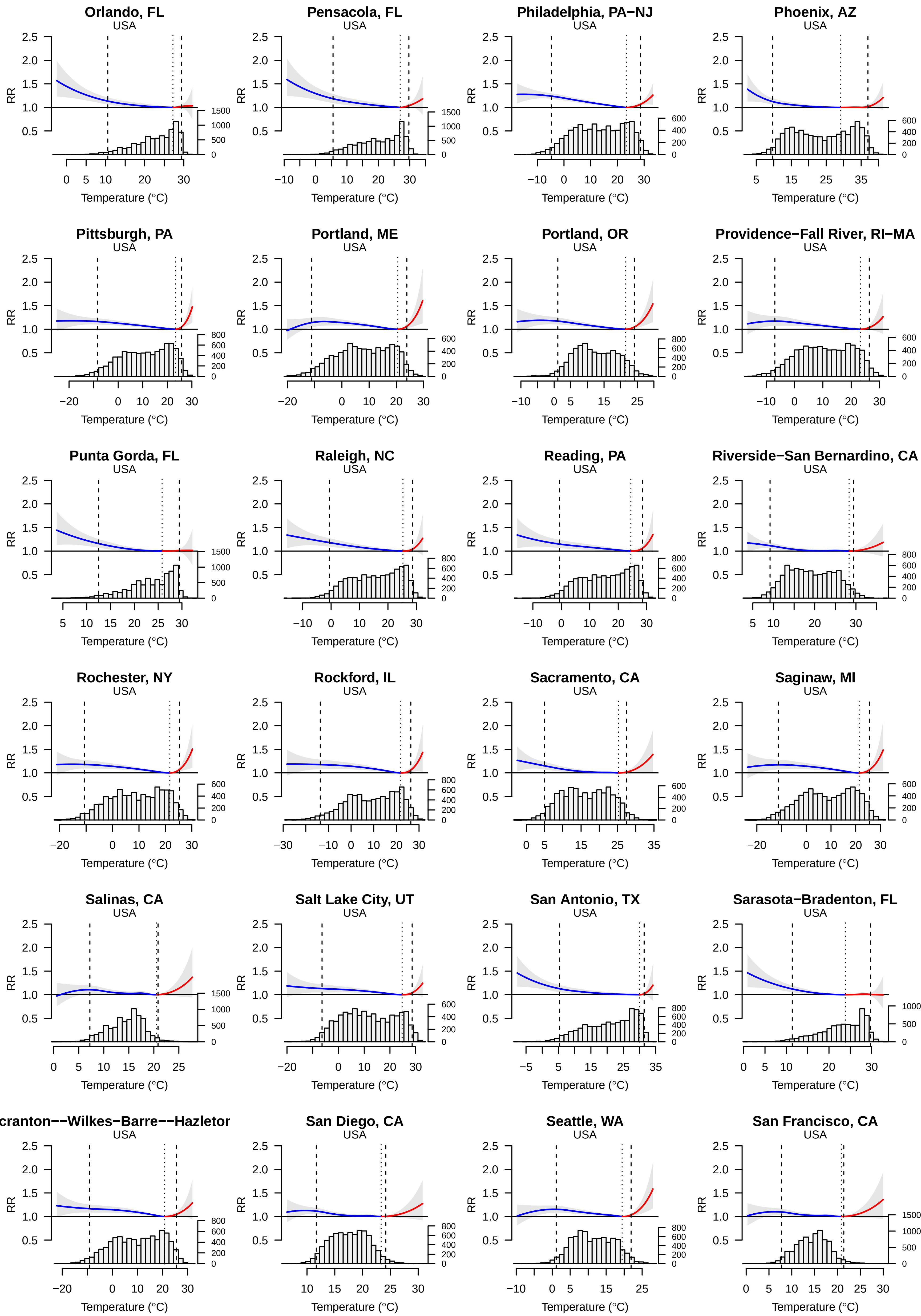

Orlando, FL
USA
Pensacola, FL
USA
Philadelphia, PA−NJ
USA
Phoenix, AZ
USA
Pittsburgh, PA
USA
Portland, ME
USA
Portland, OR
USA
Providence−Fall River, RI−MA
USA
Punta Gorda, FL
USA
Raleigh, NC
USA
Reading, PA
USA
Riverside−San Bernardino, CA
USA
Rochester, NY
USA
Rockford, IL
USA
Sacramento, CA
USA
Saginaw, MI
USA
Salinas, CA
USA
Salt Lake City, UT
USA
San Antonio, TX
USA
Sarasota−Bradenton, FL
USA
cranton−−Wilkes−Barre−−Hazletor
USA
San Diego, CA
USA
Seattle, WA
USA
San Francisco, CA
USA
RR
Temperature (°C)

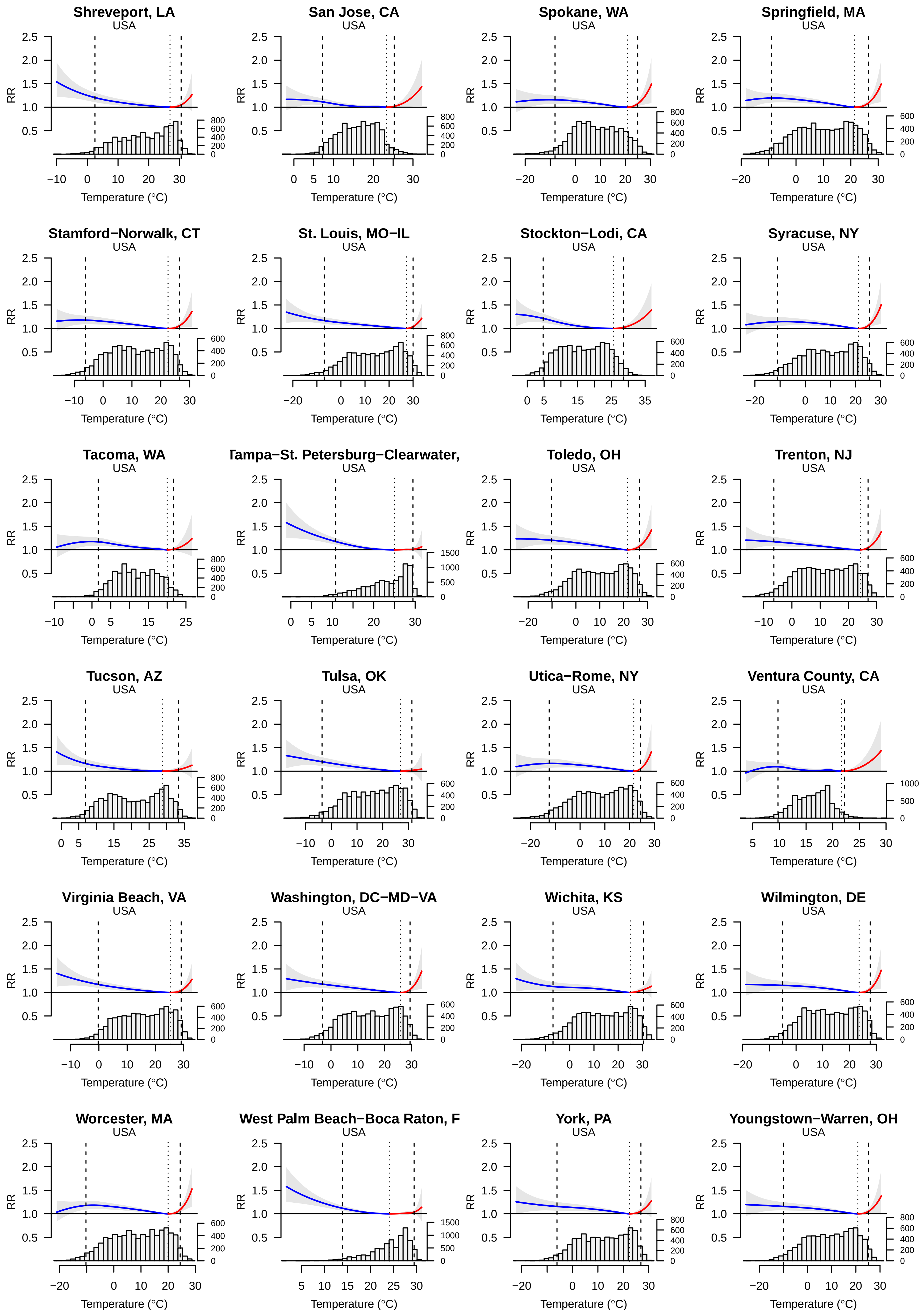
Shreveport, LA
USA
San Jose, CA
USA
Spokane, WA
USA
Springfield, MA
USA
Stamford−Norwalk, CT
USA
St. Louis, MO−IL
USA
Stockton−Lodi, CA
USA
Syracuse, NY
USA
Tacoma, WA
USA
Tampa−St. Petersburg−Clearwater,
USA
Toledo, OH
USA
Trenton, NJ
USA
Tucson, AZ
USA
Tulsa, OK
USA
Utica−Rome, NY
USA
Ventura County, CA
USA
Virginia Beach, VA
USA
Washington, DC−MD−VA
USA
Wichita, KS
USA
Wilmington, DE
USA
Worcester, MA
USA
West Palm Beach−Boca Raton, F
USA
York, PA
USA
Youngstown−Warren, OH
USA
RR
Temperature (°C)

References

1. Tong S, Wang XY, Guo Y. Assessing the short-term effects of heatwaves on mortality and morbidity in Brisbane, Australia: comparison of case-crossover and time series analyses. *PloS One* 2012; **7**(5): e37500.
2. Tong S, Wang XY, Yu W, Chen D, Wang X. The impact of heatwaves on mortality in Australia: a multicity study. *BMJ Open* 2014; **4**(2): e003579.
3. Kaplan GG, Tanyingoh D, Dixon E, et al. Ambient ozone concentrations and the risk of perforated and nonperforated appendicitis: a multicity case-crossover study. *Environmental Health Perspectives* 2013; **121**(8): 939-43.
4. Martin SL, Cakmak S, Hebbern CA, Avramescu ML, Tremblay N. Climate change and future temperature-related mortality in 15 Canadian cities. *International Journal of Biometeorology* 2012; **56**(4): 605-19.
5. Michelozzi P, de'Donato FK, Bargagli AM, et al. Surveillance of summer mortality and preparedness to reduce the health impact of heat waves in Italy. *International Journal of Environmental Research and Public Health* 2010; **7**(5): 2256-73.
6. Schifano P, Leone M, De Sario M, et al. Changes in the effects of heat on mortality among the elderly from 1998--2010: results from a multicenter time series study in Italy. *Environ Health* 2012; **11**(1): 58.
7. Tobias A, Armstrong B, Zuza I, Gasparrini A, Linares C, Diaz J. Mortality on extreme heat days using official thresholds in Spain: a multi-city time series analysis. *BMC Public Health* 2012; **12**(1): 133.
8. Rocklov J, Forsberg B, Meister K. Winter mortality modifies the heat-mortality association the following summer. *European Respiratory Journal* 2008; **33**(2): 245-51.
9. Rocklv J, Ebi K, Forsberg B. Mortality related to temperature and persistent extreme temperatures: a study of cause-specific and age-stratified mortality. *Occupational and Environmental Medicine* 2011; **68**(7): 531-6.
10. Armstrong BG, Chalabi Z, Fenn B, et al. The association of mortality with high temperatures in a temperate climate: England and Wales. *Journal of Epidemiology and Community Health* 2011; **65**(4): 340-5.
11. Gasparrini A, Armstrong B, Kovats S, Wilkinson P. The effect of high temperatures on cause-specific mortality in England and Wales. *Occupational and Environmental Medicine* 2012; **69**(1): 56-61.
12. Zanobetti A, O'Neill MS, Gronlund CJ, Schwartz JD. Susceptibility to mortality in weather extremes: effect modification by personal and small-area characteristics. *Epidemiology* 2013; **24**(6): 809-19.
13. Zanobetti A, Schwartz J. The effect of fine and coarse particulate air pollution on mortality: a national analysis. *Environmental Health Perspectives* 2009; **117**(6): 898-903.
14. Gasparrini A, Leone M. Attributable risk from distributed lag models. *BMC Medical Research Methodology* 2014; **14**(1): 55.
